# Coal falling trajectory and strength analysis of drum of shearer based on a bidirectional coupling method

**DOI:** 10.1038/s41598-024-60262-9

**Published:** 2024-04-24

**Authors:** Meichen Zhang, Lijuan Zhao, Baisheng Shi

**Affiliations:** 1 College of Mechanical Engineering, Changshu Institute of Technology, Suzhou, 215500 People’s Republic of China; 2 College of Mechanical Engineering, Liaoning Technical University, Fuxin, 123000 People’s Republic of China; 3 The State Key Lab of Mining Machinery Engineering of Coal Industry, Liaoning Technical University, Fuxin, People’s Republic of China; 4 Liaoning Province Large Scale Industrial and Mining Equipment Key Laboratory, Fuxin, 123000 Liaoning Province People’s Republic of China

**Keywords:** Bidirectional coupling method, Drum, Coal falling trajectory, Strength, Mechanical engineering, Coal

## Abstract

The cutting and crushing of coal and rock containing gangue is the result of the coupling effect of multiple factors. The geometric parameters of the working mechanism, the kinematic parameters of the shearer, and the physical and mechanical properties of the coal and rock to be cut all affect the cutting and crushing process of the shearer. To study the coal falling trajectory of cutting coal and rock using a spiral drum, optimal cutting parameters were obtained, efficient cutting using a spiral drum was achieved, and analysis of the coal falling trajectory and strength of the drum of a shearer based on bidirectional coupling technology was proposed based on particle discrete element contact theory and virtual prototype technology. The discrete element method multi-flexible body dynamics two-way coupling method was used to obtain cutting and interactive information about the spiral drum for a complex coal seam with gangue. The cutting conditions of the spiral drum under different cutting depths, rotational speeds, and traction speeds were determined. The movement status of coal and rock particles was monitored under different working conditions. Coal falling trajectory equations for the coal and rock particles were compiled under different working conditions, and the coal falling trajectory curve was drawn. The optimal coal loading rate was used as the measurement standard for the coal falling trajectory, and the optimal coal falling trajectory of the drum was obtained through full factor experiments. The load of the drum and pick was extracted, their stress and deformation were analyzed, and fatigue life analysis was performed on the pick with the highest stress. The results indicate that the maximum deformation occurs on the cutting teeth that are cutting hard gangue. The stress of the tooth seat is mainly concentrated at the root of the tooth seat, and its maximum equivalent stress is less than the yield limit value of the selected material. Therefore, the material selection and structural design of the drum are safe and reliable. By building a coal mining machine cutting coal and rock experimental platform and monitoring the working status of the designed spiral drum, it meets the usage requirements. Based on industrial experiments conducted underground, the measured average coal loading rate of the shearer drum was 46.31%, achieving stable operation and verifying that the designed drum of the shearer has an efficient cutting ability.

## Introduction

The reserves of thin coal seams in China are considerable, but for a long time, the mining of thin coal seams in China has not been prosperous^1^. The presence of gangue in thin coal seams complicates the structure of the coal seams^2–4^. Whether the gangue layer is close to the roof or floor, it will seriously affect the mining of coal seams, increase the ash content of the coal, and reduce the quality of the coal^5–8^. In addition, the presence of gangue increases the deformation of the drum during the cutting process, affecting the coal falling performance of the drum^9–11^. At present, the mining of thin coal seams containing gangue has become a hot research topic in China and abroad, and many scholars have conducted systematic research on this topic. Dewangan et al. conducted cutting tests on WC Co material picks and analyzed the effects of the installation angle and coal rock conditions on pick wear^12^. Labra et al. proposed a cutting theory based on the finite-discrete element method and analyzed the stress distribution of the interaction between the cutting teeth and coal rock^13^. Reid et al. analyzed the dynamic load characteristics of the cutting teeth of a thin coal seam shearer using an extended Kalman filter^14^. Hassanpour determined the relationship between the wear life of shearer cutting tools and the effective geological parameters of thin coal seams^15^. Comakli analyzed the influence of the geological conditions of thin coal seams in the Cappadocia region of Türkiye on the wear of mining machinery tools^16^. Gospodarczyk established a model for the coal cutting process of a shearer based on discrete element theory and analyzed the variation in the coal flow motion under different motion parameters of the shearer^17^. Jia Hanjie et al. developed a hybrid dynamics model using the dynamic substructure method (DSM) and verified the effect of topology optimization on reducing the misalignment of the equivalent mesh of the cutting teeth of thin coal seam shearers^18^. Liu et al.^19^ investigated the relationship between the coal cutting resistance of the drum of a thin coal seam shearer and the coal cutting parameters. Zhen et al.^20^ analyzed the reliability of key components of coal mining machines based on the finite element method. Zhang et al.^21^ studied on the vibration characteristics of the spiral drum of a coal mining machine based on a bidirectional coupling method. Chunping Ren et al. used the EDEM discrete element software to study simulated coal walls with different thicknesses of coal gangue and studied the load changes under different thicknesses of coal gangue^22^. Chunsheng Liu et al. used the EDEM discrete element particle simulation software to numerically simulate the coal mining process of a shearer. They analyzed the axial equivalent power, cutting power, and coal loading energy consumption^23^. Lijuan Zhao et al. established a coupling model between the drum and the coal seam containing gangue based on pro/engineer (Pro/E) and EDEM interface technology and conducted simulation cutting simulation^24^. Ordin used the inverse distance weighted (IDW) method to establish solid models of coal seams with different mechanical characteristics and studied their impact on the coal mining capacity of the shearer^25^.

The working conditions of a coal mining machine under complex coal seam occurrence conditions are harsh and the load is complex^26,27^. Conducting experiments at the mining site, due to the significant disturbance of data, it is difficult to accurately identify various parameters, and data collection is difficult and carries great risks^28–30^. Laboratory model experiments require the preparation of different coal rock walls. This is expensive, and it is difficult to ensure the similarity to coal mining faces with gangue^31^. The discrete element method and virtual prototyping technology can supplement or even replace the actual coal and rock cutting and crushing processes^32^. The dynamic simulation process not only provides important information that people cannot perceive in physical experiments but also enables us to conduct repeated experiments under different working conditions, greatly reducing research and development costs^33–35^.

Based on this, in this study, we adopted a combination of on-site survey sampling analysis, theoretical calculations, numerical simulations, and virtual prototype simulations to study the bidirectional coupling method of a coal seam discrete element model and spiral drum finite element model. We analyzed the motion trajectory of coal and rock particles that are efficiently cut and dropped by the drum. Based on the dynamic characteristic indicators of the coal mining machine, we studied the influences of the drum structure and kinematic parameters, as well as the physical and mechanical properties of the coal gangue, on the working performance of a coal mining machine. The theory and method of designing the working mechanism of coal mining machines that form structural evolution were determined. The results of this study provide a necessary theoretical basis for the development of an efficient and powerful cutting spiral drum tailored to land with independent intellectual property rights and excellent performance in China. In addition, this research method can also be extended to the design of the working mechanisms of full-section and partial-section tunneling machines, providing a new method and means for research on large-scale industrial and mining equipment systems under complex coal seam storage conditions. This method has broad application prospects.

## Establishment of a bidirectional coupling model for the cutting section of a shearer to cut coal walls

### Construction of a high-precision coal wall model for complex gangue

#### Establishment of particle filling technology for coal and rock layers with multiple mineral composition modes

**Figure 1:**
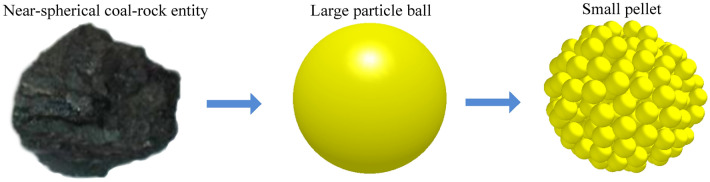
Particle benchmark model.

The shapes of coal blocks produced by mining real coal seams vary. Using a typical spherical coal and rock mass as a prototype particle model for constructing a coal wall, we can not only accurately provide effective information about the simulation results but also improve the convenience of detecting the contact state between the large particle groups and reduce the computational complexity. To achieve rapid adhesion between particles, small particle pellets were used to replace the entire large particle sphere (Fig. 1). Based on the coal and rock simulation particle radius test conducted by our research group^36^, the particle diameter of the small particle pellets was set to 12 mm.

The material structure of a coal and rock mass is complex, and its internal microstructure is generally formed via polymerization of various minerals, which are anisotropic. To improve the accuracy of the three-dimensional simulation model of a coal wall, a multiple mineral composition particle filling technology for coal and rock was developed using the EDEM discrete element secondary development function.

Based on Pro/E, a particle space filling model of coal and rock was constructed and imported into the analysis system (ANSYS) to divide the grid. The generated structural mesh was converted into discrete point coordinates and was exported as an msh file. The specific position of the small particle pellets in the spatial model was obtained from the X, Y, and Z coordinate information provided by the msh file. The particle material properties of the coal and rock were set, and a reasonable range of parameter changes was utilized. The material parameter values obtained using small particle pellets at different locations within the same coal and rock particles were different. This broke down the solidization of the materials that generate coal and rock particles, making them similar to the actual structure and composition of coal and rock materials. It was assumed that the number of small particle pellets generated in the space filling model was *Y*, and the filling end time of all of the small particle pellets in the space was *m*. They were arranged in the chronological order in which the small particle pellets were generated *b*, and were output in the form of row vectors. The expression is as follows:

1 $$N_{bt} = \left( {N_{b,1}^{t} ,N_{b,2}^{t} , \cdot \cdot \cdot N_{b,m}^{t} } \right)$$

where, $N_{b,1}^{t} ,N_{b,2}^{t} , \cdot \cdot \cdot N_{b,m}^{t}$ is the location point of the small particle pellets in the space filling model.

The material parameter values that can represent the properties of the same coal and rock particles were defined as *x* groups, and the construction matrix (Eq. (2)) represents the positional order of the small particle pellets arranged within the range of the complete rest time *t*:

2 $$NU_{t} \in Q^{x \times m}$$

The matrix *NU*_*t*_ is replicated *L* times to obtain $NU_{t}{^\prime} \in Q^{{\left( {x \times L} \right) \times m}}$, which is expressed as follows:

3 $$NU_{t}{^\prime} = \left[ {\begin{array}{*{20}c} {NU_{t} } \\ {NU_{t} } \\ \cdot \\ \cdot \\ \cdot \\ {NU_{t} } \\ \end{array} } \right]_{{\left( {x \times L} \right) \times m}}$$

where, *L* = [*W*/*x*]. By changing the numerical value of *L*, the small particle pellets randomly match the material parameter values, and the search location coordinates automatically fill the entire space filling model.

**Figure 2:**
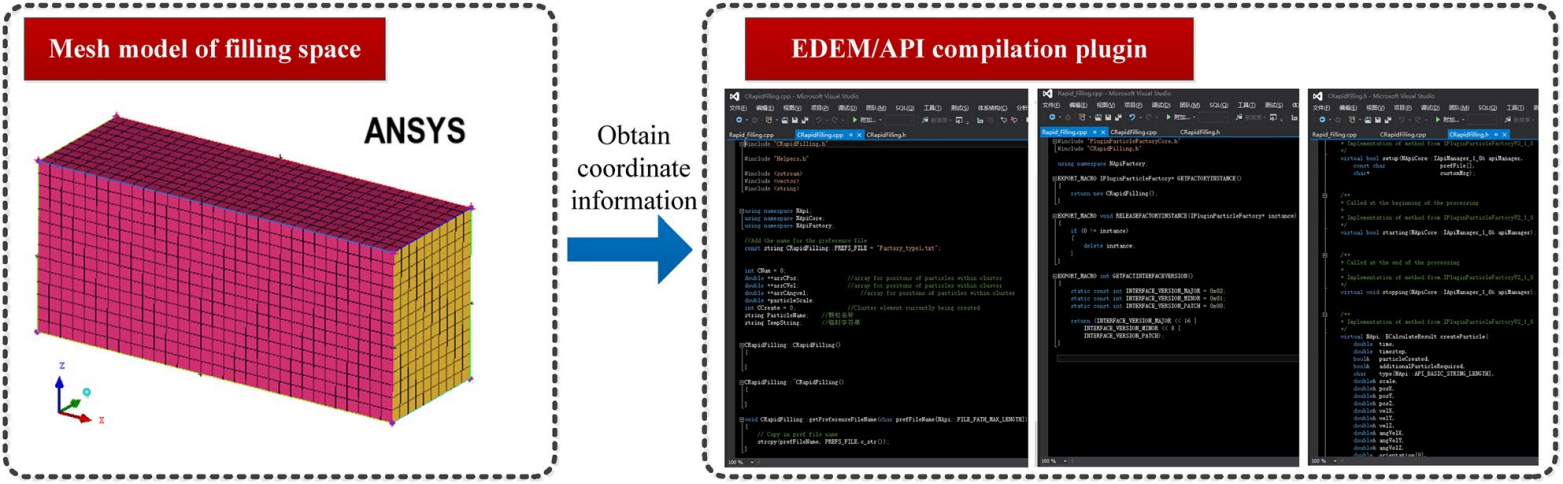
Procedure for particle filling part of the pure coal seam.

Due to the current inability of EDEM to meet the demand for particle random distribution matching, the EDEM/application programming interface (EDEM/API) function was used to write the location and material matching information about the small particle pellets into the source file of the particle factory. After reading the coordinate information, the small particle pellets match the material and automatically find the location in the filled space. Taking the pure coal seam as an example, the particle filling process is shown in Fig. 2.

#### Construction of custom contact model for a coal and rock mass

The actual surface of a coal and rock mass is characterized by unevenness, and it is difficult to fully characterize the occlusal action between the coal and rock particles by only relying on the friction force between the particles defined in the Hertz–Mindlin contact model. Therefore, based on this, the torsional force between the coal and rock particles was added, and the surface roughness was simulated by constructing a custom contact model. In the custom contact model for a coal wall, the contact unit consists of three parts: tangential, normal, and rotational.

The tangential contact force between the middling coal and rock particles in the custom contact model was calculated based on the incremental superposition method of the Mindlin theory. In addition, tangential sliding friction was added to the model, and its judgment criterion was based on the Mohr–Coulomb friction law. The mathematical model can be expressed as follows:

4 $$f_{h}^{z + 1} = f_{h}^{z} + K_{h} \mu \Delta t = f_{h}^{z} + \left( {\frac{E}{1 + \mu }} \right)^{\frac{2}{3}} \frac{{\left( {12\left( {1 - \mu } \right)R^{\prime } F_{n} } \right)^{\frac{1}{3}} }}{2 - \mu }\mu \Delta t$$

5 $$\begin{array}{*{20}c} {F_{h}^{z + 1} = f_{h}^{z + 1} - j_{h} \mu } & {if\left| {m_{h}^{z + 1} } \right| < \upsilon_{\omega } R^{\prime } \left| {F_{h}^{z + 1} } \right|} \\ \end{array}$$

6 $$\begin{array}{*{20}c} {F_{h}^{z + 1} = sign\left( {f_{h}^{z + 1} } \right)\upsilon_{h} \left| {F_{h1}^{z + 1} } \right|} & {if\left| {m_{j}^{z + 1} } \right| < \upsilon_{\omega } R^{\prime } \left| {F_{h}^{z + 1} } \right|} \\ \end{array}$$

where *f*_*h*_^*z*+1^ is the estimated initial value of the force between the tangential contacts of the particles at time *t*^*z*+1^ (N); *f*_*h*_^*z*^ is the estimated initial value of the force between the tangential contacts of the particles at time *t*^*z*^ (N); *K*_*h*_ is the tangential stiffness between the particle contacts (N/m); *j*_*h*_ is the tangential viscous damping coefficient between the particle contacts (N.s/m); *v*_h_ is the tangential static sliding friction coefficient between the particle contacts; *F*_*h*_^*z*+1^ is the force between the particles in tangential contact at time *t*^*z*+1^ (N);* E* is the elastic modulus of the particle (MPa); *μ* is Poisson’s ratio of the particles; and *R*^*′*^ is the contact radius between the particles in contact (mm).

The normal contact force of coal and rock particles was calculated based on Hertz theory through the amount of overlap between them (Eqs. (7)–(9)):

7 $$F_{nr} = K_{n} l_{n} = \vartheta \frac{E}{{\left( {1 - \mu^{2} } \right)}}\left( {R^{\prime } } \right)^{\frac{1}{2}} l_{n}$$

8 $$F_{nv} = - j_{n} \mu$$

9 $$F_{n} = F_{nr} + F_{nv}$$

where *F*_*nr*_ is the normal elastic contact force between the particle contacts (N); *F*_*nv*_ is the normal viscous retardation force between the particle contacts (N); *F*_*n*_ is the normal resultant force between the particle contacts (N); *K*_*n*_ is the normal contact stiffness between the particle contacts (N/m); *ϑ* is the force coefficient between the normal contacts of the particles; *l*_*n*_ is the amount of contact overlap between the particles in contact (mm); and *j*_*n*_ is the normal viscous damping coefficient between the particle contacts (N.s/m).

The rotational force between the coal and rock particles was calculated based on a torsional spring rolling mechanics model. In addition, a selector for Coulomb’s law was added to the model to determine whether the contact type between the particles was rotational friction resistance. The mathematical model for the anti-turning moment is as follows:

10 $$m_{\omega }^{z + 1} = m_{\omega }^{z} - K_{\omega } (\omega_{A} - \omega_{B} )\Delta t$$

11 $$\begin{array}{*{20}c} {M_{\omega }^{z + 1} = m_{\omega }^{z + 1} - j_{\omega } (\omega_{A} - \omega_{B} )} & {if\left| {m_{\omega }^{z + 1} } \right| < \upsilon_{\omega } R^{\prime } \left| {F_{n}^{z + 1} } \right|} \\ \end{array}$$

12 $$\begin{array}{*{20}c} {M_{\omega }^{z + 1} = sign(m_{\omega }^{z + 1} )j_{\omega } R^{\prime } \left| {F_{n}^{z + 1} } \right|} & {if\left| {m_{\omega }^{z + 1} } \right| < \upsilon_{\omega } R^{\prime } \left| {F_{n}^{z + 1} } \right|} \\ \end{array}$$

where $m_{\omega }^{z + 1}$ is the estimated initial value of the anti-rotation bending moment between the particle contacts at *t*^*z*+1^(N); $m_{\omega }^{z}$ is the estimated initial value of the anti-rotation bending moment between the particle contacts at time *t*^*z*^ (N); *K*_ω_ is the rotational stiffness between the particles in contact (N/m); *ω*_*A*_ and *ω*_*B*_ are the rotational speeds of particles A and B in contact (rad/s), respectively; $M_{\omega }^{z + 1}$ is the anti-rotation bending moment between the particle contacts at time *t*^*z*+1^ (N.m); $j_{\omega }$ is the viscous damping coefficient when rolling friction occurs between the particles in contact (N.s/m); and $\upsilon_{\omega }$ is the static friction damping coefficient when rolling friction occurs between the particles in contact (N.s/m).

In addition, the rotational stiffness of the coal and rock particles was determined using the anti-rotation coefficient and the normal contact stiffness (Eq. (13))^37^:

13 $$K_{\omega } = \alpha_{b}^{2} \left( {\overline{{R^{\prime } }} } \right)^{2} K_{n}$$

where $\overline{{R^{\prime } }}$ is the average radius of the contact particles (mm); and *α*_*b*_ is the anti-rotation coefficient.

#### Discrete element model for a coal wall with complex gangue

**Figure 3:**
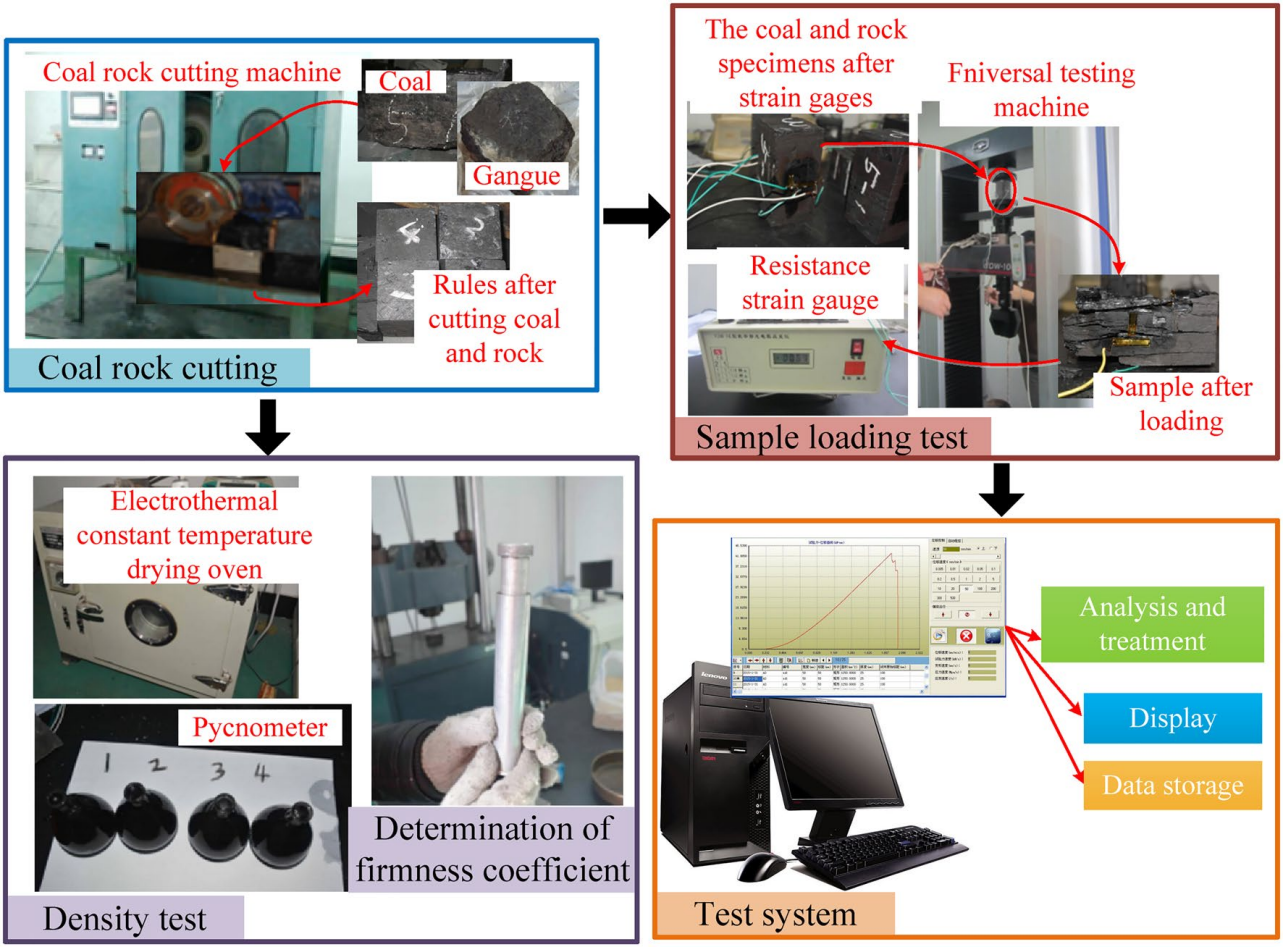
Tests of the physical and mechanical parameters of coal and rock.

According to the occurrence conditions of the coal seams in the Yanzhou Mining Area, sampling tests were conducted on the coal seams in the mining area according to sampling principles and detection standards. The relevant experiments are shown in Fig. 3, and Table 1 presents the specific physical and mechanical performance parameters of the coal-rock obtained from the experiments.

**Table 1:** Physical and mechanical property parameters of the coal and rock.

Coal seam distribution	Lithology	Density (kg/m^3^)	Modulus of elasticity (GPa)	Poisson's ratio (*μ*)	Tensile strength (MPa)	Firmness coefficient (*f*)
17 top coal	17 coal	1.31e3	5.24	0.31	1.73	2.38
Hard gangue	limestone	2.63e3	12.1	0.23	3.76	5.1
17 medium coal	17 coal	1.31e3	5.24	0.31	1.73	2.38
gangue	Aluminous mudstone	2.46e3	3.62	0.24	1.19	3.5
17 bottom coal	17 coal	1.31e3	5.24	0.31	1.73	2.38

**Figure 4:**
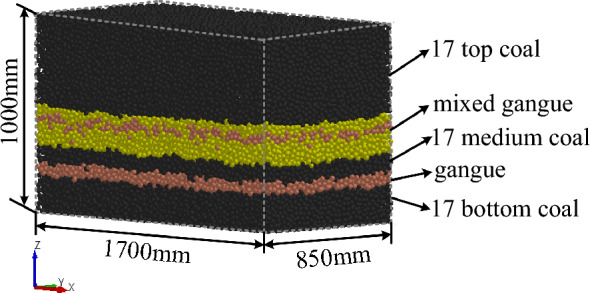
Discrete element model of coal wall with complex gangues.

Based on the physical and mechanical performance parameters of the coal-rock obtained during the experiment (Fig. 3), a discrete element model of a coal wall with complex gangue was constructed using particle filling technology for simulating multi-mineral composition coal and rock layers and the calculation technology for a custom contact model for coal and rock mass. The final established discrete element model of a high-precision coal wall with complex gangues is shown in Fig. 4.

#### Verification of the experiment

**Figure 5:**
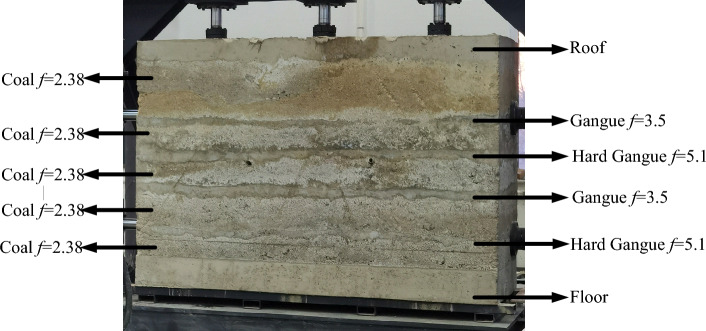
Actual coal wall model.

To verify that the performance of the coal wall obtained using the high-precision coal wall model construction method described above is close to that of real coal rock, in the experiment, we prepared a coal wall with a length of 2000 mm, a height of 1500 mm, and a thickness of 800 mm in the Key Laboratory of Liaoning Province using large-scale industrial and mining equipment. The entire coal wall was composed of a roof, floor, coal seam, gangue layer, and hard gangue layer. The final simulated experimental coal wall is shown in Fig. 5.

**Figure 6:**
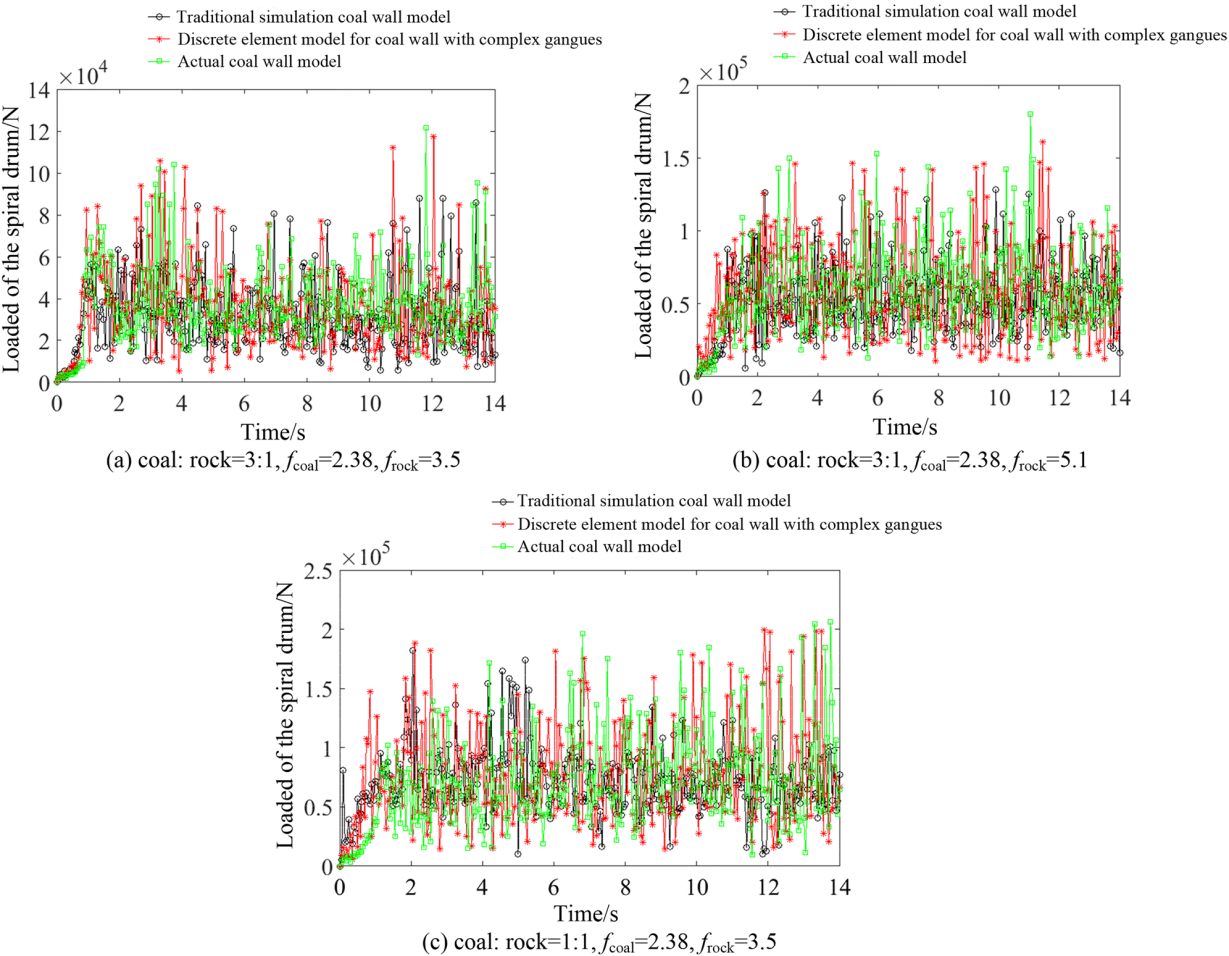
Experimental comparison results.

We conducted cutting experiments using the completed coal wall, and we set the traction speed of the shearer to 0.07 m/s and the drum rotation speed to 9.5 rad/s to obtain the force of the spiral drum during the cutting process. The working conditions of the coal were as follows: rock = 3:1, *f*_coal_ = 2.38, *f*_rock_ = 3.5, coal: rock = 3:1, *f*_coal_ = 2.38, *f*_rock_ = 5.1, coal: rock = 1:1, *f*_coal_ = 2.38, and *f*_rock_ = 3.5. The traditional coal wall^38^ and the high-precision complex gangue coal wall construction method proposed in this article were used to establish their corresponding working conditions for coal walls. The experimental results were compared with the force of the spiral drum of the traditional simulation coal wall model and the high-precision discrete element model of the coal wall (Fig. 6).

As can be seen from Fig. 6, the average load and load fluctuation coefficient of the drum under the construction method of the high-precision discrete element model of a coal wall are improved compared with the simulation results of cutting traditional simulation coal wall models. The load on the drum under the three working conditions increased by 14.76%, 9.89%, and 8.74%, and the load fluctuation coefficient increased by 20.13%, 14.37%, and 16.24%. During the process of cutting the high-precision discrete element model of the coal wall, the error between the load on the drum and the actual coal rock is smaller. This indirectly verifies that the performance of the discrete element simulation model for a coal wall with gangue constructed in this study is closer to the actual coal-rock mass.

### Constructing a virtual prototype model of rigid flexible coupling for a shearer cutting section

The Pro/E software was used to establish rigid models of various parts of the cutting section of the shearer, and then we performed non-interference assembly. The cutting part assembly was imported into the RecurDyn in stp * format, the quality of each part was defined, and constraints and drivers were added based on the actual working principle of the cutting part. The two important parts in the process of establishing a virtual prototype model for rigid flexible coupling of the cutting section of a shearer are the calculation of the contact and the generation of the flexible parts.

#### Calculation of contact

The contact type of solid to solid in RecurDyn used the contact analysis algorithm of the relative coordinate system^39^, and the calculation process is as follows:

14 $$F = K\delta + C\overrightarrow {v}$$

where *δ* is the penetration depth (mm); *K* is the stiffness of the contact (N/mm); $\vec{v}$ is the relative velocity of the contact point (mm/s); and *C* is the contact damping (${\text{N}} \cdot {\text{S/mm}}$).

It can be seen from Eq. (14) that the parameters, such as the penetration depth, contact damping, and contact stiffness, need to be effectively set during the process of adding contact to the gear transmission system. The penetration depth can be obtained by solving the contact parts using the finite element method. The contact damping between the contact gears is calculated as follows:

15 $$C_{\max } = 14 \times 10^{9} \frac{{E_{t} }}{{2.1 \times 10^{11} }}w_{t} s_{t}$$

16 $$C_{\min } = \gamma_{t} C_{\max }$$

where *E*_*t*_ is the elastic modulus of the contact part (GPa); *w*_*t*_ is the effective contact area between the parts in contact (mm^2^); *s*_*t*_ is the deformation coefficient of the contact part; and *γ*_*t*_ is the stiffness ratio of the parts in contact.

The contact stiffness between the contact gears is calculated as follows:

17 $$K_{t} = \sum\limits_{i = 1}^{2} {\left( {\frac{1}{{\frac{1}{{k_{h,i} }} + \frac{1}{{k_{b1,i} }} + \frac{1}{{k_{f1,i} }}}} + \frac{1}{{\frac{1}{{k_{h,i} }} + \frac{1}{{k_{b2,i} }} + \frac{1}{{k_{f2,i} }}}}} \right)}$$

18 $$K_{h} = \sum\limits_{i = 1}^{2} {\left\{ {\frac{{4\left[ {\left( {1 - \mu_{1,i}^{2} } \right)E_{2} + (1 - \mu_{2,i}^{2} )E_{1} } \right]}}{{3\pi^{2} E_{1,i} E_{2,i} }} \times \left[ {\frac{{R_{1,i} R_{2,i} }}{{R_{1,i} + R_{2,i} }}} \right]^{\frac{1}{2}} } \right\}}$$

19 $$\frac{1}{{K_{b} }} = \int_{{ - \beta_{1} }}^{{\beta_{2} }} {\left[ {\frac{{3{ + }3cos\beta_{1} \times \left[ {\left( {\beta_{2} - \beta_{1} } \right)sin\alpha_{l} - cos\alpha_{l} } \right]^{2} (\beta_{2} - \alpha_{l} )cos\alpha_{l} }}{{2EB_{l} \left[ {sin\alpha_{l} + \left( {\beta_{2} - \alpha_{l} } \right)\cos \alpha_{l} } \right]^{3} }}} \right]}$$

20 $$\frac{1}{{K_{f} }} = \frac{{\cos^{2} \alpha_{l} }}{{EB_{l} }}\left\{ {l\left( {\frac{{w_{f} }}{{b_{f} }}} \right)^{2} + m\left( {\frac{{w_{f} }}{{b_{f} }}} \right) + n\left( {1 + p\tan^{2} \alpha_{l} } \right)} \right\}$$

where *K*_*h*_ is the Hertz contact stiffness, *K*_*b*_ is the gear bending stiffness, and the gear deformation stiffness *K*_*f*_ constitutes the total meshing stiffness of the gear contact *K*_*t*_ (N/mm); *μ* is Poisson’s ratio of the contact gear; *R* is the contact radius between the contacting gears (mm); *B*_*l*_ is the tooth width of the gear (mm); *α*_*l*_ is the pressure angle of the gear (°); *β*_1_ is the residual angle of the included angle between the tooth profile center line of the contact gear and the tooth tip involute (°); *β*_2_ is half of the included angle of the tooth profile involute of the contact gear forming an arc on its base circle (°); *w*_*f*_ is the distance from the intersection point between the tooth shape center line of the contact gear and the curvature line of the initial meshing point to the tooth root circle (mm); *b*_*f*_ is the tooth shape width of the contact gear on the tooth root circle (mm); and *l*, *m*, *n*, and *p* are the effective coefficients for the contact stiffness deformation.

#### Generation of flexible parts

**Figure 7:**
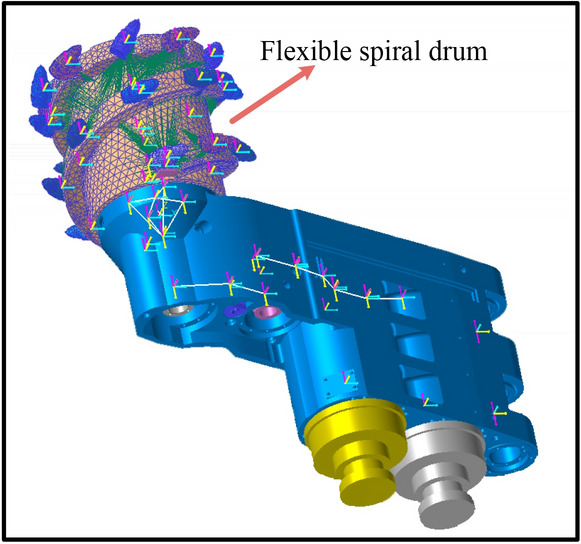
The rigid-flexible coupling virtual prototype model of the cutting section of the shearer.

Due to the fact that the flexibility of the components can greatly reduce the speed of the simulation, to obtain accurate coal-falling trajectory information while improving the feasibility of the implementation of the simulation, we selected the components that directly affect the trajectory information during the cutting of the cutting section to achieve flexibility. During the cutting and crushing process, the spiral drum is in direct contact with the coal wall, and the high impact it receives and its own parameters directly affect the coal-falling trajectory of the coal-rock. Therefore, the spiral drum was subjected to flexible processing. Finally, the rigid-flexible coupling virtual prototype model of the cutting section of the shearer was constructed (Fig. 7).

### Establishment of a bidirectional coupling model for DEM-MFBD in the cutting section

#### Bidirectional coupling model interaction process

**Figure 8:**
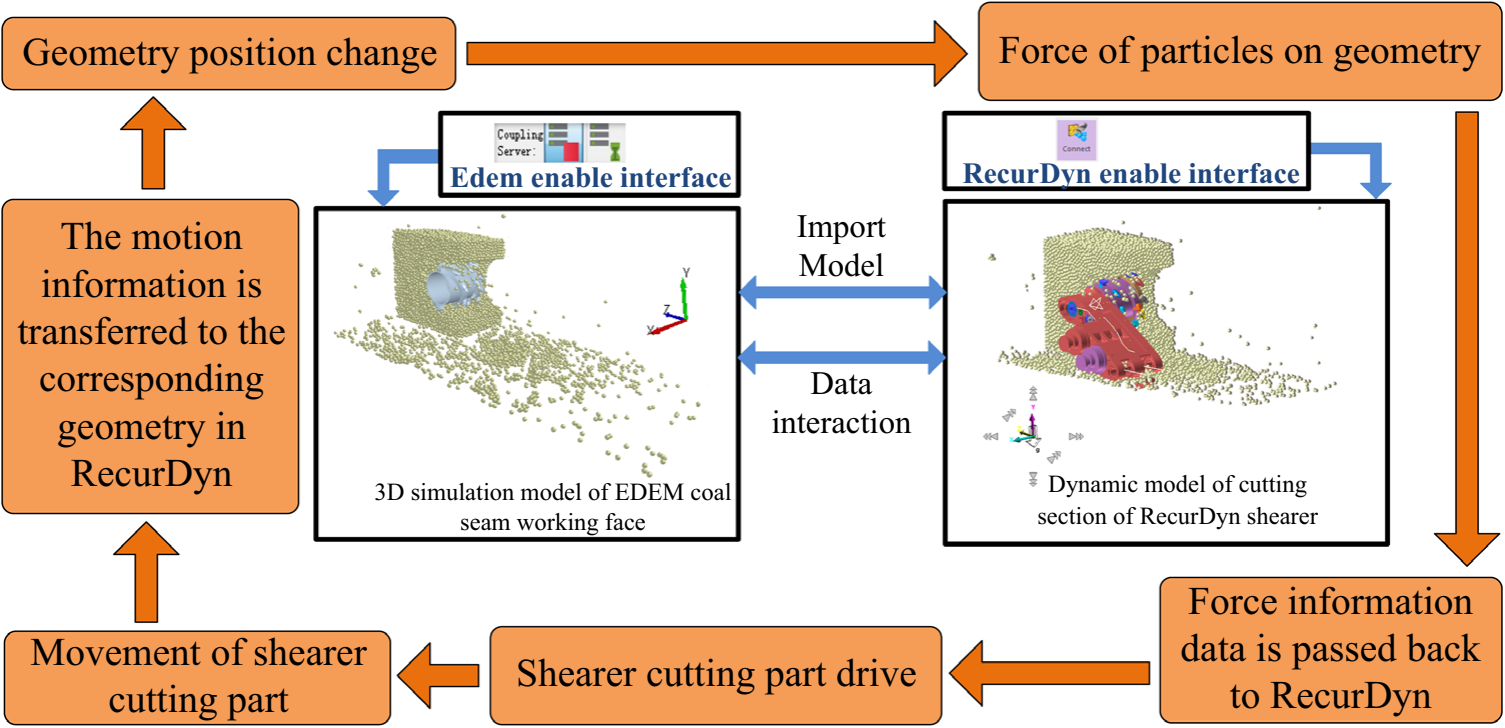
The bidirectional coupling interaction process.

The bidirectional coupling model of the cutting process of the shearer’s cutting section was established through the coupling interface in EDEM-RecurDyn, yielding the association between the discrete element model of the coal wall with complex gangue and the rigid-flexible coupling virtual prototype model of the cutting section. The bidirectional coupling interaction process is shown in Fig. 8. As shown in Fig. 8, RecurDyn transmits the translational and rotational motion information of the dynamic model of the cutting section of the shearer to the corresponding geometric body in EDEM. The change in the position of the geometric body leads to changes in the position, direction, and magnitude of the force on the working face of the coal seam. EDEM calculates the force exerted by the coal wall on the geometry at this time and transmits the data back to RecurDyn. At the beginning of the next time step, RecurDyn calculates new motion information based on the feedback load information and internal driving information and transmits it to EDEM, achieving real-time bidirectional transmission of the load and motion displacement data during the interaction between the coal wall simulation model and the cutting part of the shearer.

#### Model comparison verification

**Figure 9:**
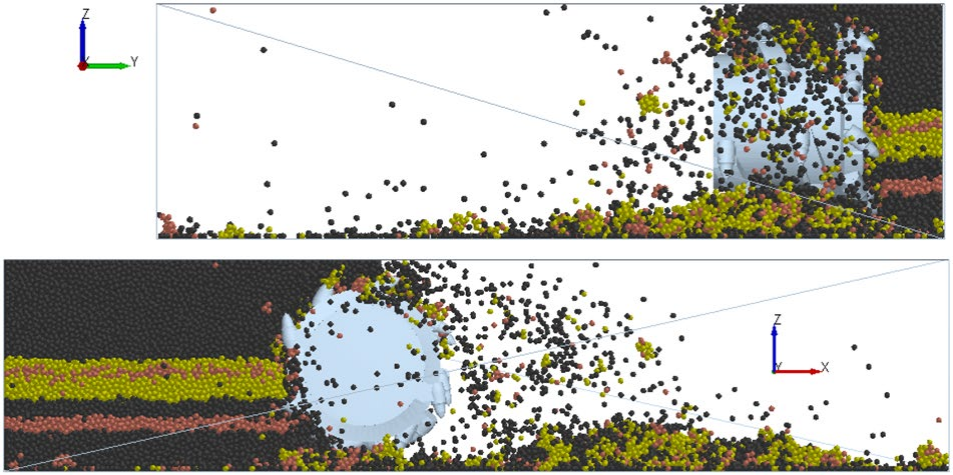
The cutting process.

**Figure 10:**
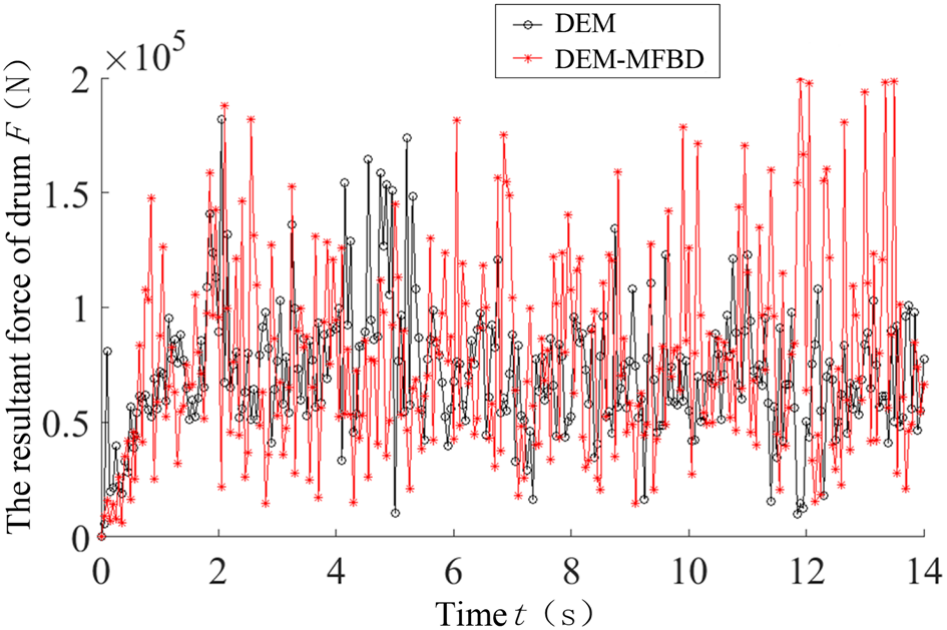
The resultant force curve of the spiral drum.

The cutting process of the shearer’s cutting section was simulated at a rotational speed of 9.5 rad/s and a traction speed of 0.07 m/s. The cutting process is shown in Fig. 9. The resultant force curve of the spiral drum under these working conditions was plotted (Fig. 10).

As can be seen from Fig. 10, after the spiral drum comes in contact with the coal wall, the stress increases sharply and there is a significant nonlinear change. There are differences in the changes between the bidirectional and unidirectional coupling. During the bidirectional coupling process, the resultant force on the drum under these working conditions is 77,628.9772 N, and the load fluctuation coefficient is 0.5619. Under these working conditions, the resultant force on the drum during unidirectional coupling is 71,388.1874 N, and the load fluctuation coefficient is 0.4834. The resultant force and load fluctuation coefficient of the drum under the bidirectional coupling simulation are both improved compared with the unidirectional coupling simulation results, and the drum load is increased by 8.74% and the load fluctuation coefficient is increased by 16.24%.

To verify the accuracy of the DEM-MFBD bidirectional coupling model, laboratory experiments were conducted to obtain the three-way force on the spiral drum under these working conditions, and the results were compared with the results for bidirectional coupling and unidirectional coupling. The results are compared in Table 2.

**Table 2:** The comparison results.

Force on spiral drum (N)	Experimental result	Error with DEM-MFBD (%)	Error with DEM (%)
Traction resistance direction	36,417.3229	1.17	7.78
Direction of lateral force	8717.6191	1.87	7.09
Cutting resistance direction	63,721.4196	2.46	8.11
Three-way resultant force	79,316.6572	2.12	9.97

As can be seen from Table 2, the three-dimensional force of the drum obtained from the laboratory experiments is basically consistent with the DEM-MFBD bidirectional coupling numerical simulation results. Due to the fact that the simulated coal wall is a uniform coal-rock mass composed of particle bonding and the experimental coal wall is made by mixing gypsum, cement, and other raw materials, there are varying degrees of errors in the results. The simulation results of the discrete element method (DEM) unidirectional coupling numerical simulation differ greatly from the laboratory experiment results, with a maximum error of 9.97%. The error between the simulation results of the DEM-MFBD bidirectional coupling numerical simulation and the experimental measurements is smaller than the simulation results of the DEM unidirectional coupling numerical simulation, so the bidirectional coupling numerical simulation model is more consistent with the actual working state of the spiral drum.

## Simulation and result analysis of falling-coal trajectory of the cutting of a coal wall containing gangue using a spiral drum

### Simulation conditions

The trajectory of the coal-rock particles after separation from the coal wall is affected by three factors. 1) The mechanical action between the cutting picks and the stripped coal-rock particles changes its motion trajectory. The cutting picks cut the coal wall so that the coal-rock particles obtain an initial velocity. At this time, some of the particles are directly thrown by the cutting picks to the goaf via parabolic motion. The selection of the kinematic parameters of the spiral drum that provides the power source for the coal-rock particles and the size of the collision recovery coefficient between the cutting picks that play the main role of cutting and the coal-rock particles are important factors that determine the initial speed of the coal-rock particles. The kinematic parameters of the spiral drum can be controlled manually, and the collision recovery coefficient between the cutting picks and the coal-rock particles can be determined using its own material. 2) The coal-rock particles not thrown by the cutting pick are squeezed out of the coal wall by the spiral blade. Therefore, the structural parameters of the spiral blade and the mechanical action between the spiral blade and the coal-rock particles also change the trajectory of the particles. 3) Whether the coal-rock particles are thrown out by the cutting picks or squeezed out by the spiral blade, once they collide with other particles in the air, their motion trajectory also changes.

Therefore, through the above analysis, under the condition that the structural parameters, material parameters, and physical and mechanical properties of the spiral drum are unchanged, the effects of the changes in the spiral drum’s traction speed, rotation speed, and cutting depth on the movement trajectory of the coal-rock particles were analyzed, and the coal loading rate under different working conditions was determined for verification. Table 3 presents the 15 working conditions set based on the single factor method.

**Table 3:** The working conditions.

Type	Working condition	Traction speed/*v* (m/s)	rotating speed/*n* (rad/s)	cutting depth/*b* (mm)
A	1	0.07	9.5	430
	2	0.07	9.5	480
	3	0.07	9.5	530
	4	0.07	9.5	580
	5	0.07	9.5	630
B	1	0.07	7.5	530
	2	0.07	8.5	530
	3	0.07	9.5	530
	4	0.07	10.5	530
	5	0.07	11.5	530
C	1	0.03	9.5	530
	2	0.05	9.5	530
	3	0.07	9.5	530
	4	0.09	9.5	530
	5	0.11	9.5	530

### Determination of falling-coal trajectory based on discrete element simulation

After the coal-rock particles leave the spiral drum, in the subsequent movement process, although some of the particles collide with each other again, thus changing the falling-coal trajectory, they account for a small part and can be ignored. Therefore, the following important assumptions were made. When the coal-rock particles fall off the coal wall and are no longer in contact with the spiral drum, the coal-rock particles only undergo simple parabolic movement in the air. Then, the general expression of the three-dimensional parabolic curve of the coal-rock particle trajectory was derived:

21 $$Z = ax^{2} + by^{2} + c$$

where *Z*, *x*, and *y* are the position coordinates of the coal and rock particles, *a* and *b* are the coefficients of the trajectory equation, and *c* is the cutting radius of the drum.

**Figure 11:**
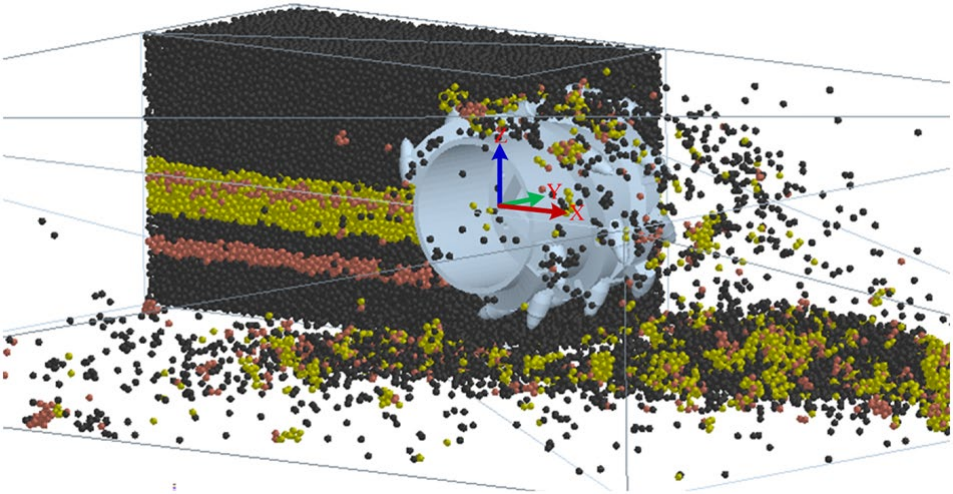
The establishment of three-dimensional coordinate system in EDEM.

**Figure 12:**
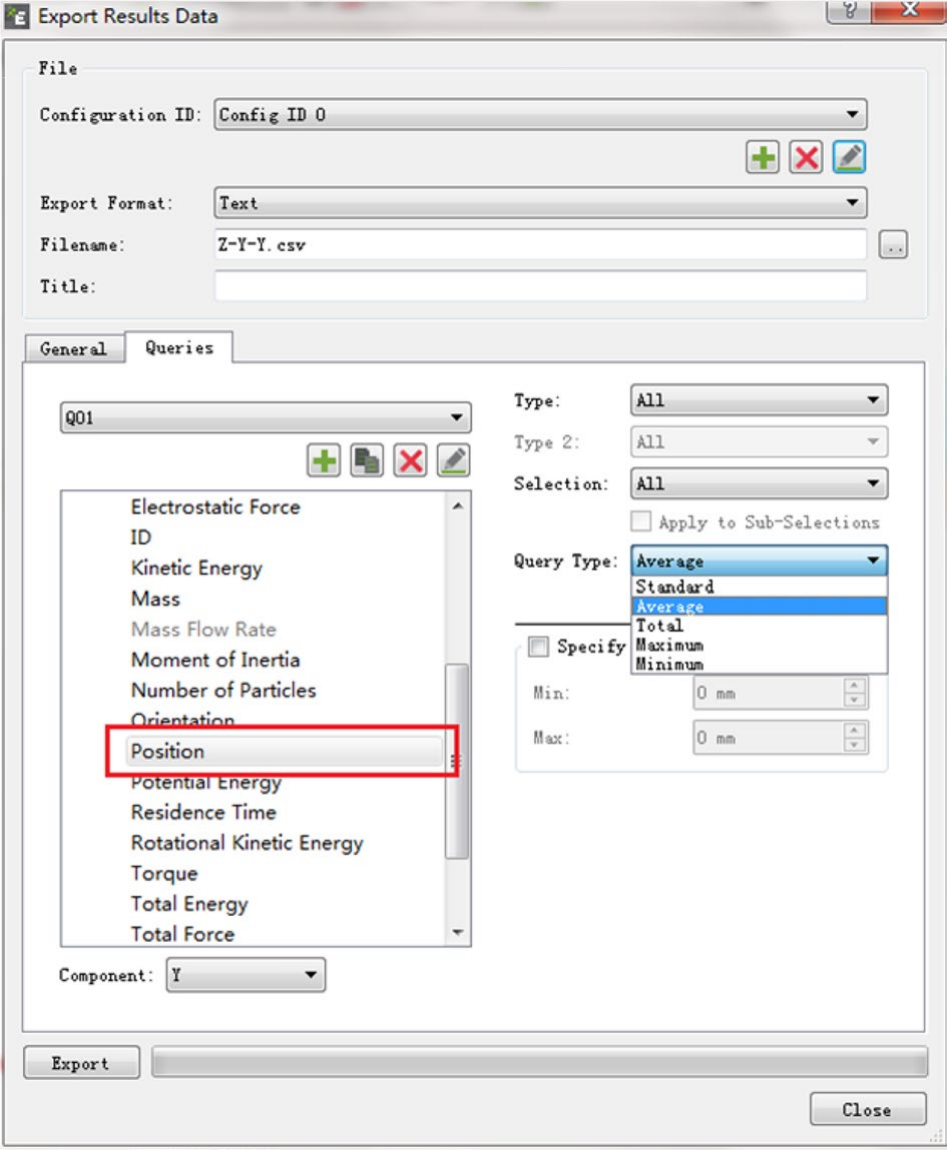
Export of post-processing data in EDEM.

With the center of mass of the spiral drum as the origin, the reverse direction of the traction speed is the X-axis, the cutting depth direction is the Y-axis, and the vertical upward direction is the Z-axis, and thus, a three-dimensional coordinate system is established (Fig. 11). According to the average position of the moving particles in space, the parabolic equation of the falling-coal trajectory of the coal-rock particles was solved. In addition, the convergence trajectory curve of the coal-rock particles under different conditions was fitted using MATLAB. The position coordinates of the moving particles can be derived through post-processing in EDEM. The specific operation is shown in Fig. 12.

### Analysis of factors influencing the falling-coal trajectory

#### Influence of the change of the cutting depth of the spiral drum on the falling-coal trajectory

**Figure 13:**
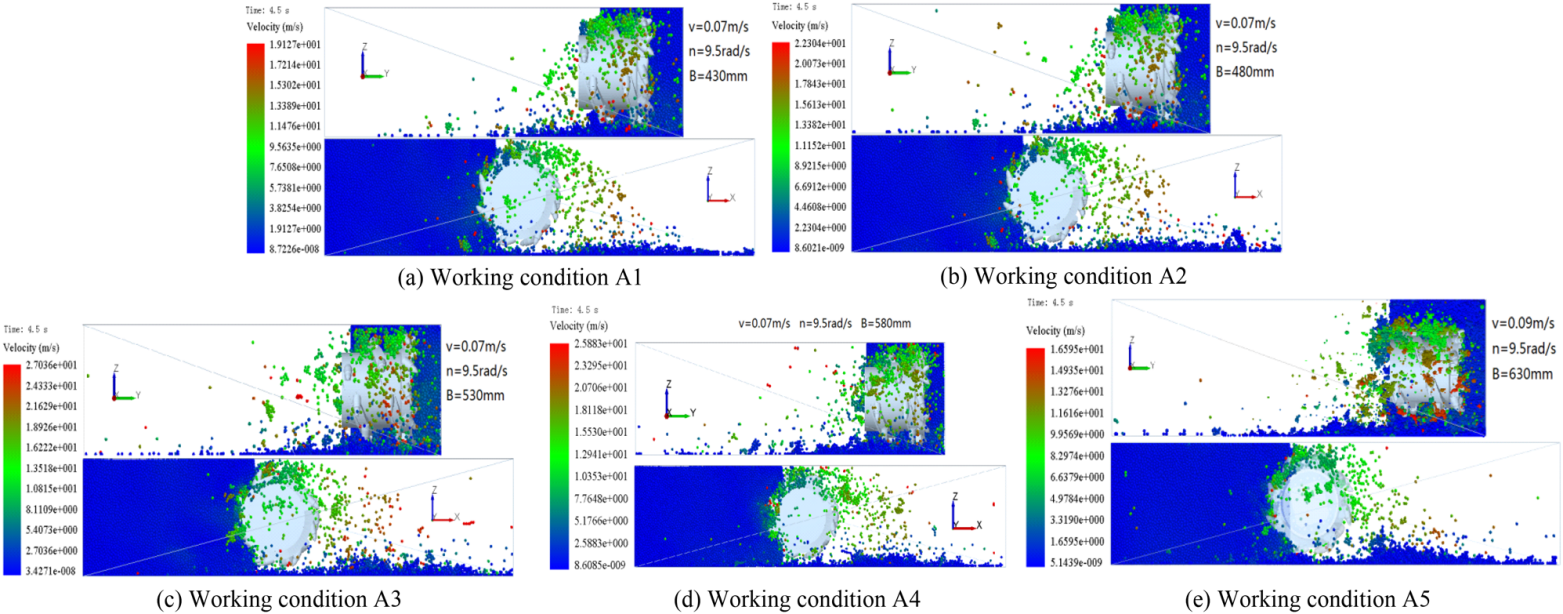
The particle velocity cloud of the coal-rock under different cutting depth.

When the traction speed of the shearer is 0.07 m/s, the rotation speed of the spiral drum is 0.09 rad/s, and the cutting depths of the spiral drum is 430, 480, 530, 580, and 630 mm, the cloud diagram of the falling-coal track speed at the same position on the coal wall being cut is shown in Fig. 13.

**Figure 14:**
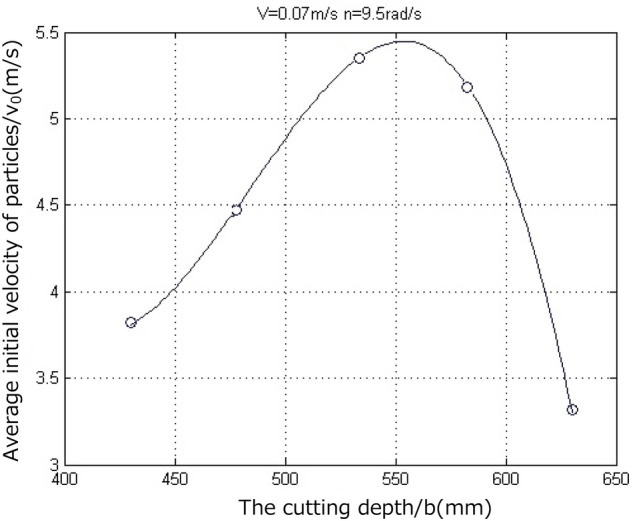
Average initial velocity of the coal-rock particles at different cutting depth.

As can be seen from the velocity cloud diagram in Fig. 13, for the cutting depths of the spiral drum listed above, the average initial velocities of the coal-rock particles were calculated to be 3.8254, 4.4608, 5.4073, 5.1766, and 3.3190 m/s. As the distance between the coal-rock particles and the ground surface increases, the velocity of the coal-rock particles becomes faster and faster under the action of gravitational acceleration. At this time, when the cutting depths are 430, 480, 530, 580, and 630 mm, the maximum speeds of the coal rock particle peeling are 19.127, 22.304, 27.036, 25.883, and 16.596 m/s, respectively. Some special coal rock particles achieve higher speeds because they are crushed by the tip of the cutting teeth during the drum cutting process. We analyzed the X and Y direction views of the velocity nephogram and determined that as the cutting depth increases, the number and average velocity of the coal-rock particles in the area far from the drum initially increase and then decrease. Regarding the simulation results for a cutting depth of 530 mm, the area far from the drum has the largest number and average speed of the coal-rock particles. In summary, when the rotation speed and the shearer traction speed of the spiral drum are constant, as the drum cutting depth continuously increases, the initial speed obtained by stripping the coal-rock particles initially increases and then decreases (Fig. 14). As a result, the maximum velocity and maximum displacement of the particles also initially increase and then decrease.

**Figure 15:**
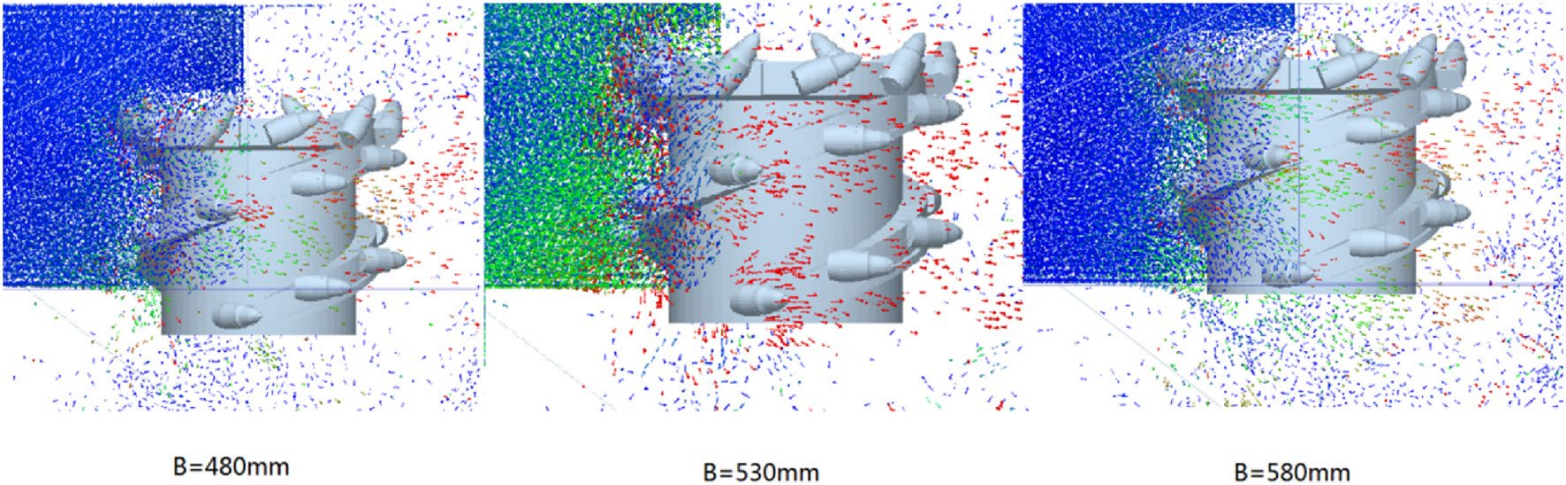
Velocity vector diagrams of the coal-rock particles under different cutting depth conditions.

Although the velocity nephogram can clearly show the velocity and displacement of the coal-rock particles, it is difficult to see the velocity direction of the coal-rock particles in the process of parabolic movement. Therefore, the velocity vector diagram for the process of coal and rock particle movement was exported. The velocity vector diagrams for the three working conditions and for cutting depths of 480, 530, and 580 mm were selected for analysis (Fig. 15).

It can be seen from Fig. 15 that the number of coal particles that changed their initial velocity direction was the largest when the cutting depth was 530 mm. That is, the spiral blade responsible for coal squeezing and coal loading played the largest role at this time. By observing the coal-rock particles in the uncut coal wall, it was observed that most of the particles in the uncut coal wall were green under the cutting depth of 530 mm. This shows that a large part of the coal-rock particles in the coal wall to be cut exhibited a movement trend, which was conducive to the next coal falling process of the spiral drum. However, the movement trends of the uncut coal wall particles in the coal walls with cutting depths of 480 and 580 mm were not obvious.

Based on the velocity nephogram and velocity vector diagram of the coal-rock particles, according to the method presented in Section "Analysis of factors influencing the falling-coal trajectory", we selected the average value of the coordinates of the special position points of the coal-rock particles when the spiral drum cuts the same position of the coal wall under different cutting depths (Table 4).

**Table 4:** Position coordinates of coal and rock particles under different cutting depth conditions.

Working condition	Z1	X	Y	Z2	X	Y
A1	0.200754	0.5312870	0	− 0.274584	0	− 0.252451
A2	0.216939	0.5488104	0	− 0.288245	0	− 0.263733
A3	− 0.245156	0.5898954	0	− 0.293872	0	− 0.302312
A4	− 0.207969	0.5041115	0	− 0.311622	0	− 0.313438
A5	− 0.189976	0.4916332	0	− 0.326983	0	− 0.315546

According to the values of X, Y, and Z in Table 4, the convergence parabolic curve equation of the falling-coal trajectory of the coal-rock particles under different cutting depths was solved using Eq. (22):

22 $$\left\{ \begin{gathered} Z_{1} = - 0.1164x_{1}^{2} - 10.895y_{1}^{2} + 0.4 \hfill \\ Z_{2} = - 0.6078x_{2}^{2} - 9.8457y_{2}^{2} + 0.4 \hfill \\ Z_{3} = - 1.8540x_{3}^{2} - 7.5922y_{3}^{2} + 0.4 \hfill \\ Z_{4} = - 2.3924x_{4}^{2} - 7.2435y_{4}^{2} + 0.4 \hfill \\ Z_{5} = - 5.8924x_{5}^{2} - 6.6435y_{5}^{2} + 0.4 \hfill \\ \end{gathered} \right.$$

**Figure 16:**
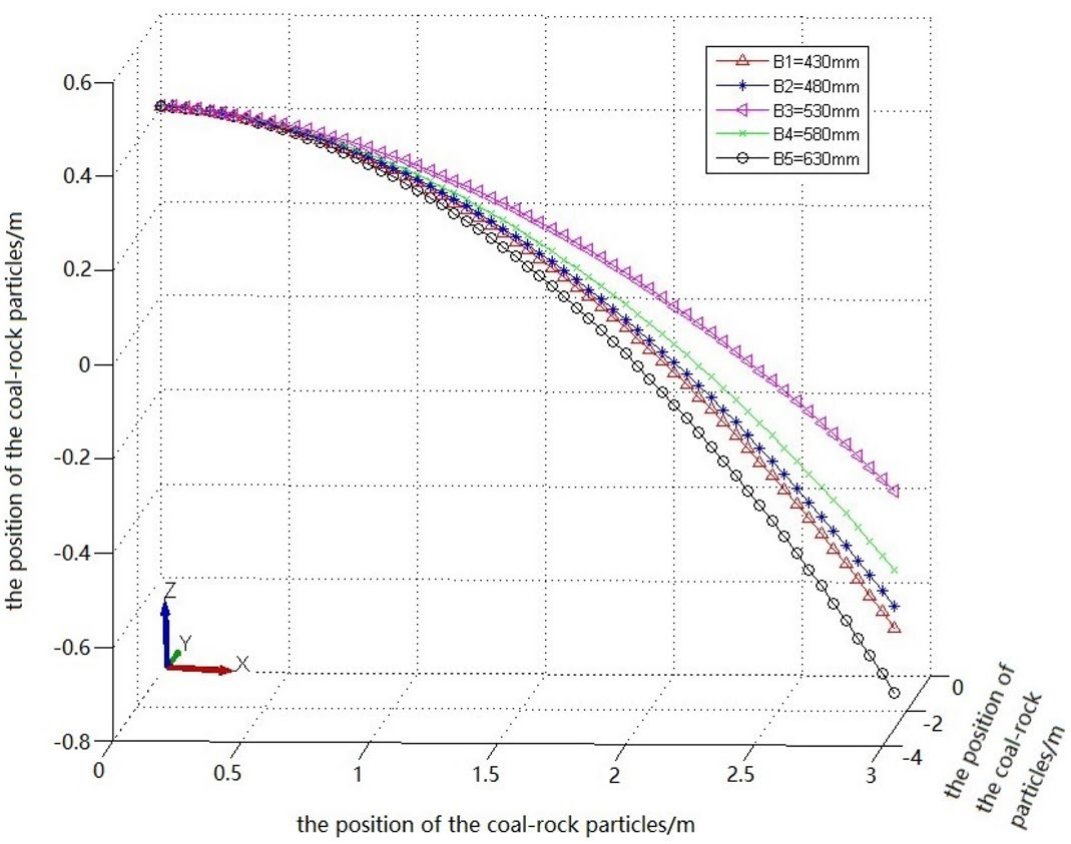
The trajectory of falling-coal in different cutting depths.

According to the fitted falling-coal trajectory equation, the falling-coal trajectory curves under different cutting depth conditions were drawn using MATLAB (Fig. 16).

It can be seen from the curve in Fig. 16 that as the cutting depth increased, the opening of the parabolic curve of the falling-coal trajectory initially increased and then decreased. That is, as the cutting depth of the spiral drum increased, the distance that the coal-rock particles were thrown initially increased and then decreased. This is consistent with the previous conclusion of the velocity nephogram of the coal-rock particles, which verifies the correctness of the trajectory of the coal-rock particles for different cutting depths.

#### Influence of the change in the rotation speed of the spiral drum on the falling-coal trajectory

**Figure 17:**
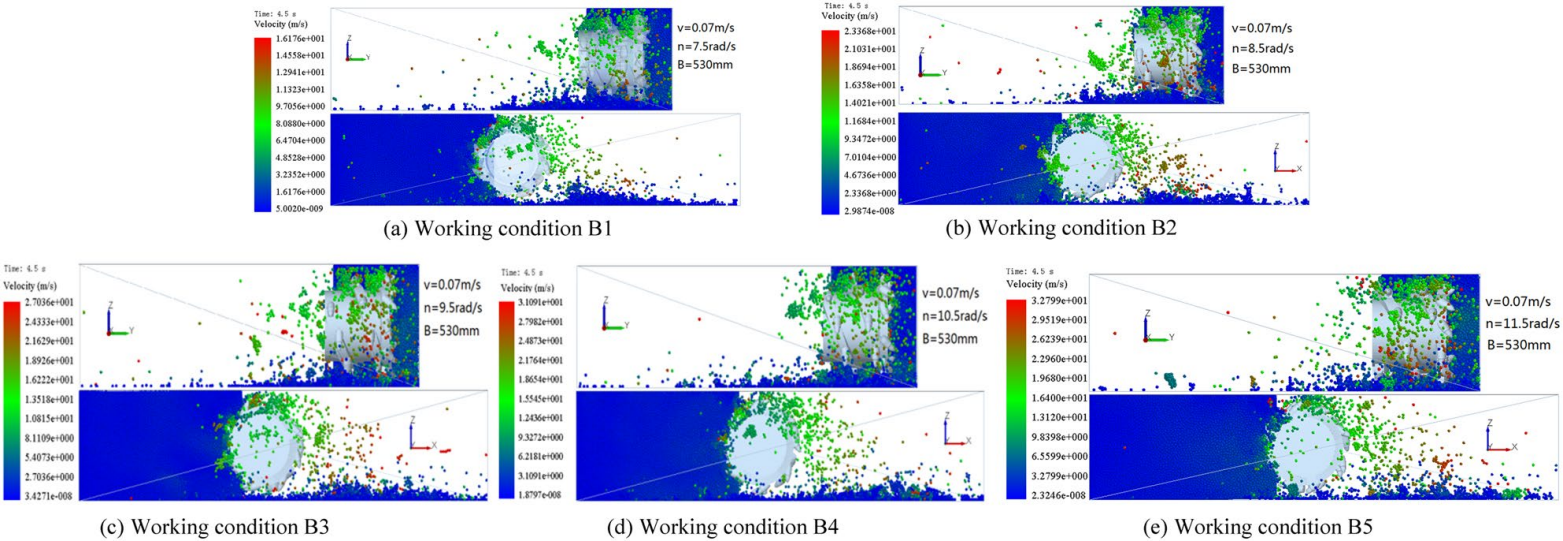
The velocity cloud of the coal-rock particles at different rotating speed.

When the traction speed of the shearer is 0.07 m/s, the cutting depth of the spiral drum is 530 mm, and the rotation speeds of the spiral drum are 7.5, 8.5, 9.5, 10.5, and 11.5 rad/s, a cloud diagram of the falling-coal track speed at the same position on the cut coal wall is shown in Fig. 17.

**Figure 18:**
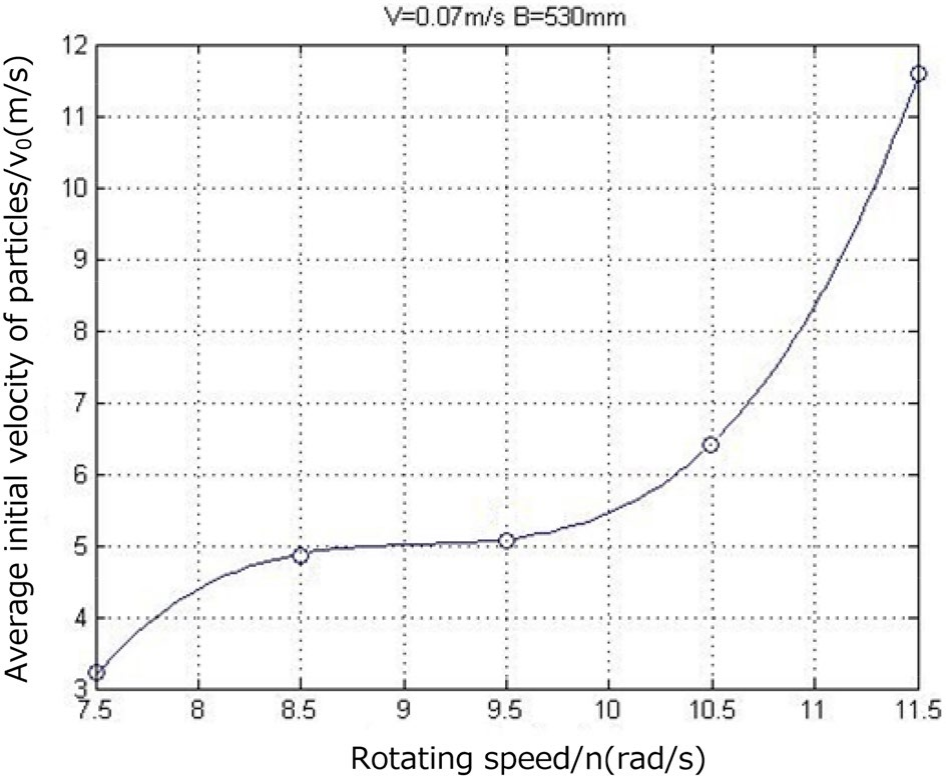
Average initial velocity of coal and rock particles at different rotating speed.

As can be seen from the velocity cloud diagram in Fig. 17, when the rotation speeds are 7.5, 8.5, 9.5, 10.5, and 11.5 rad/s, the initial velocity ranges of the coal-rock particles stripped by the spiral drum are 1.6176–4.8528, 2.3368–7.0104, 2.7036–8.1109, 3.1091–9.3272, and 3.2799–9.8398 m/s, respectively. Thus, the average initial velocities of the coal-rock particles are calculated to be 3.2352, 4.6736, 5.4073, 6.2181, and 6.5599 m/s, and the maximum velocities of the stripped coal-rock particles are 16.176, 23.368, 27.036, 31.091, and 32.799 m/s, respectively. We analyzed the X and Y direction views of the velocity nephogram and found that as the rotation speed increased, the number and average velocity of the coal-rock particles in the area far from the drum increased. In summary, when the cutting depth of the spiral drum and the shearer traction speed are constant, as the spiral drum rotation speed increases, the initial speed obtained by the stripped coal-rock particles increases (Fig. 18). As a result, the maximum velocity and maximum displacement of the particles also gradually increase.

**Figure 19:**
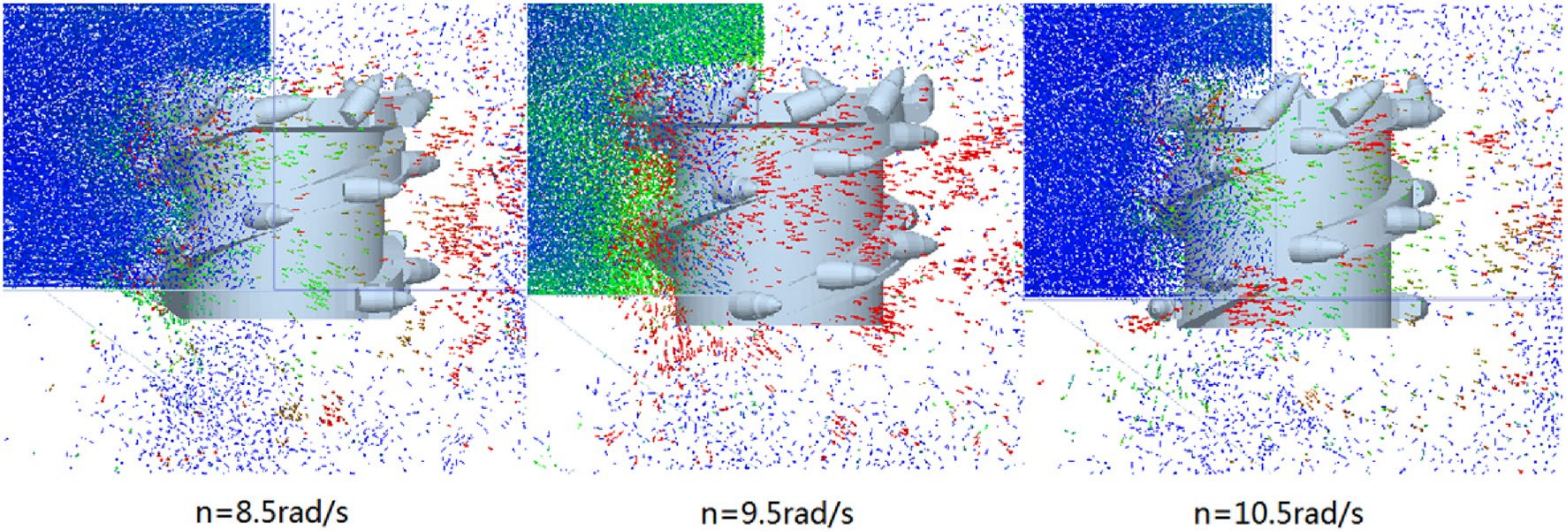
Velocity vector diagrams of the coal-rock particles under different rotating speed conditions.

Similarly, the velocity vector diagram during the movement of the coal-rock particles was drawn. The velocity vector diagrams for the three working conditions and rotation speeds of 8.5, 9.5, and 10.5 rad/s were selected for analysis (Fig. 19).

As can be seen from Fig. 19, when the rotation speed was 9.5 rad/s, the number of coal particles that changed their initial velocity directions was the largest, that is, the spiral blade responsible for coal squeezing and coal loading played the largest role at this time. By observing the coal-rock particles in the uncut coal wall, it was observed that most of the particles in the uncut coal wall were green under a rotation speed of 9.5 rad/s. This shows that a large proportion of the coal-rock particles in the coal wall to be cut exhibited a movement trend, which was further conducive to the next coal falling process of the spiral drum. However, the movement trends of the uncut coal wall particles in the coal wall under rotation speeds of 8.5 and 10.5 rad/s were not obvious.

The position coordinates of the coal-rock particles under different rotation speeds were extracted(Table 5).

**Table 5:** Position coordinates of Coal and Rock particles under different rotational Speed conditions.

Working condition	Z1	X	Y	Z2	X	Y
B1	− 2.065127	0.4856973	0	− 0.245124	0	− 0.286454
B2	− 0.224452	0.5051657	0	− 0.252381	0	− 0.297234
B3	− 0.245156	0.5898954	0	− 0.293872	0	− 0.302312
B4	− 0.237127	0.5962087	0	− 0.293937	0	− 0.322555
B5	− 0.214578	0.6014578	0	− 0.278496	0	− 0.312564

By solving the parabolic equation of the coal-rock particle falling-coal trajectory using the parameters in Table 5, the following results were obtained:

23 $$\left\{ \begin{gathered} Z_{1} = - 2.5523x_{1}^{2} - 8.4316y_{1}^{2} + 0.4 \hfill \\ Z_{2} = - 2.4470x_{2}^{2} - 7.3842y_{2}^{2} + 0.4 \hfill \\ Z_{3} = - 1.8540x_{3}^{2} - 7.5922y_{3}^{2} + 0.4 \hfill \\ Z_{4} = - 1.7924x_{4}^{2} - 6.6701y_{4}^{2} + 0.4 \hfill \\ Z_{5} = - 0.6014x_{5}^{2} - 4.2623y_{5}^{2} + 0.4 \hfill \\ \end{gathered} \right.$$

**Figure 20:**
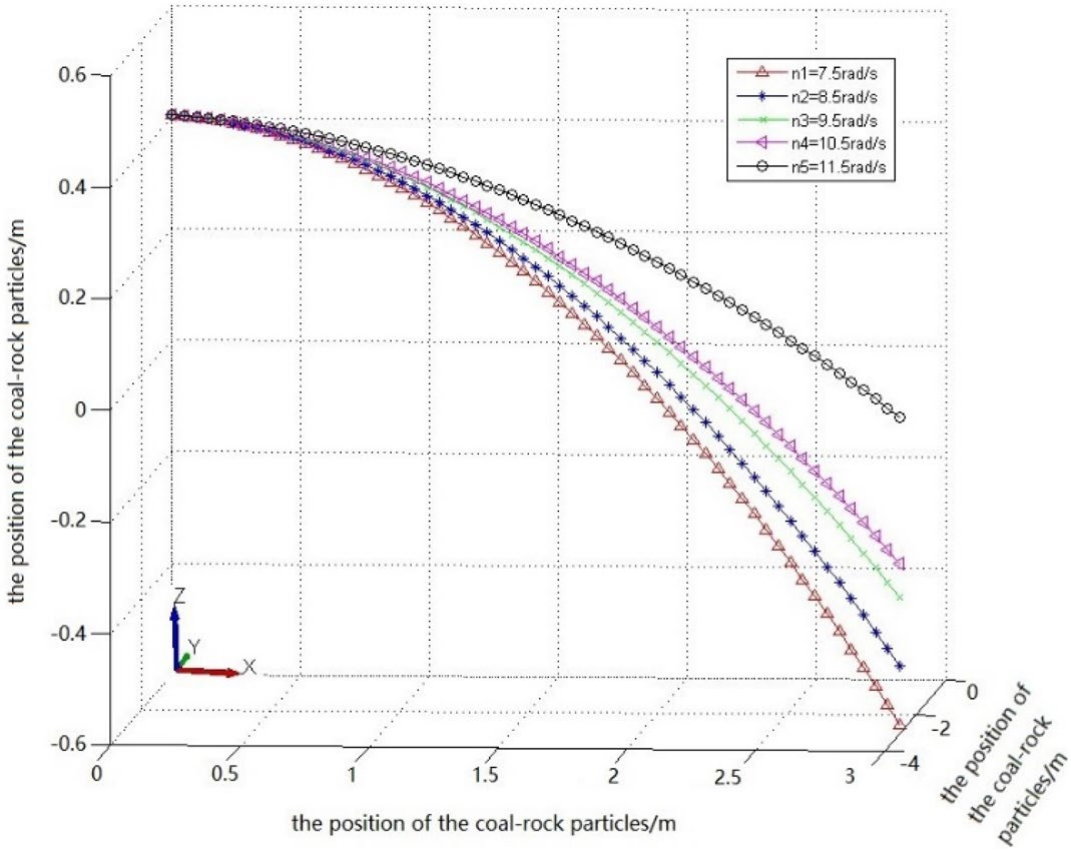
The trajectory of falling-coal in different rotating speed.

Falling-coal trajectory curves under different rotation speeds were drawn using MATLAB (Fig. 20).

It can be seen from the curve in Fig. 20 that as the rotation speed increased, the opening of the parabolic curve of the falling-coal trajectory increased. That is, as the rotation speed of the spiral drum increased, the distance that the coal-rock particles were thrown gradually increased. This is consistent with the conclusion based on the velocity nephogram of the coal-rock particles, which verifies the correctness of the trajectories of the coal-rock particles with different rotation speeds.

#### Influence of the change in the traction speed on the falling-coal trajectory

**Figure 21:**
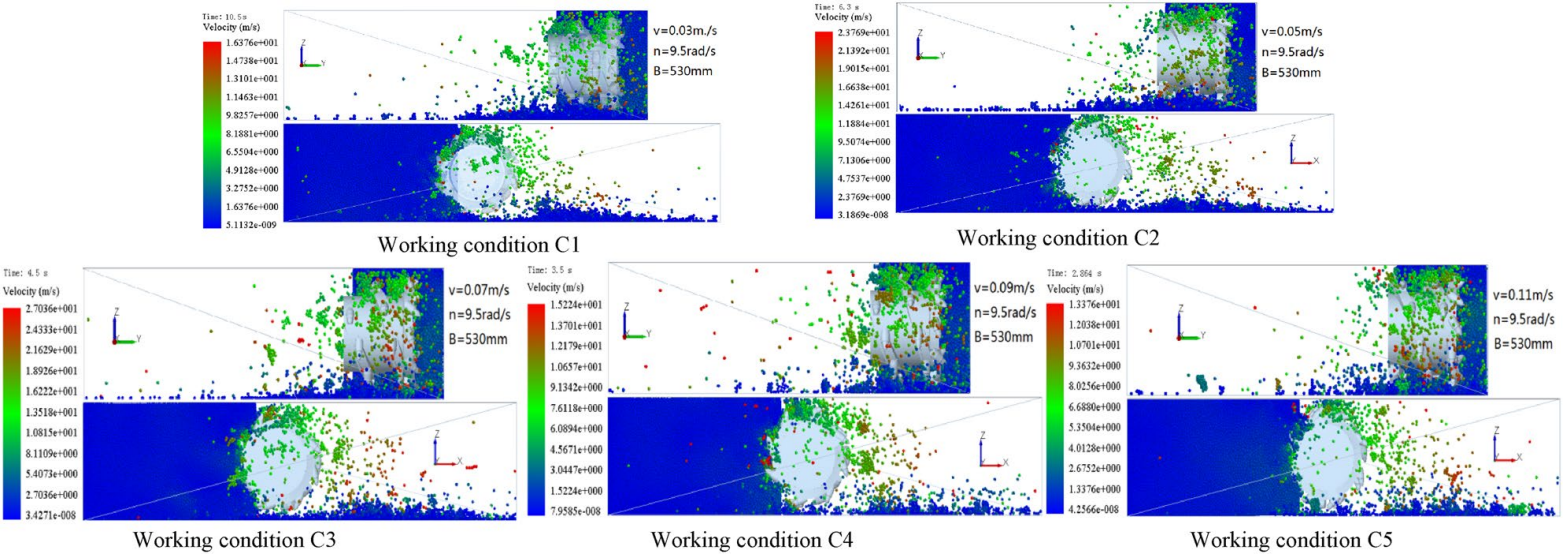
The velocity cloud of the coal-rock particles at different traction speed.

When the rotation speed of the shearer was 9.5 rad/s, the cutting depth of the spiral drum was 530 mm, and the traction speeds were 0.03, 0.05, 0.07, 0.09, and 0.11 m/s, the cloud diagram of the falling-coal track speed at the same position on the cutting coal wall was determined (Fig. 21).

**Figure 22:**
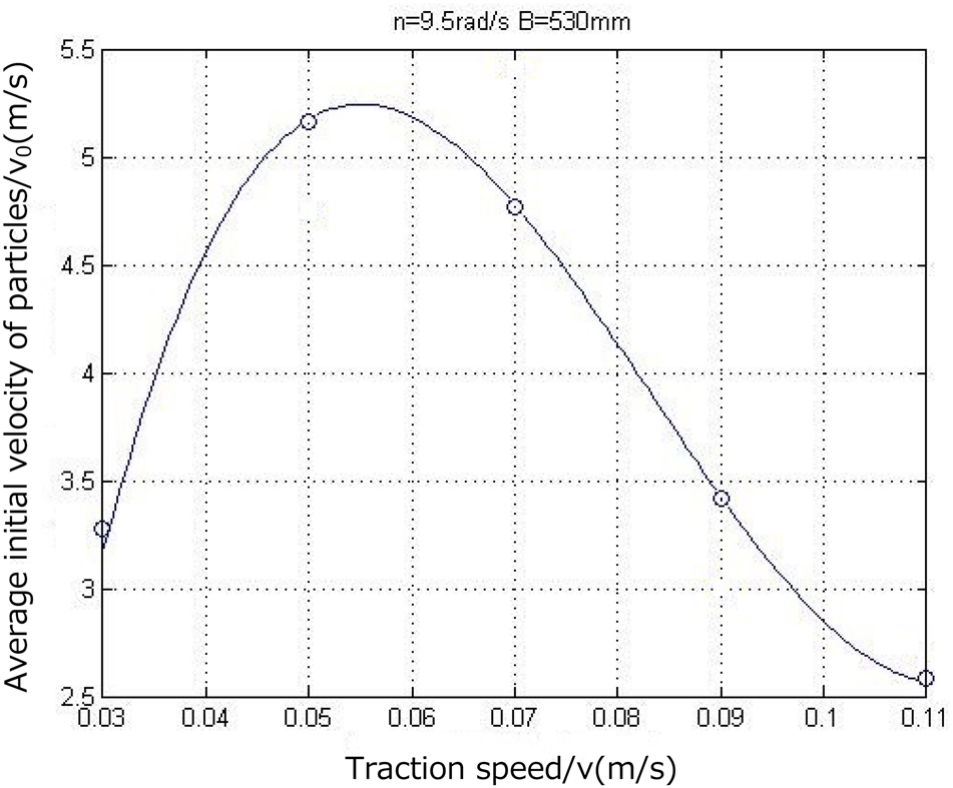
Average initial velocity of coal and rock particles at different traction speed.

According to Fig. 21, it was calculated that the average initial velocities of the coal-rock particles were 3.2752, 4.7537, 5.4073, 3.0447, and 2.6752 m/ and, the maximum velocities at which the coal-rock particles were stripped were 16.376, 23.769, 27.036, 15.224, and 13.376 m/s for the five traction speeds listed above, respectively. We analyzed the X and Y direction views of the velocity nephogram and found that as the traction speed increased, the number and average velocity of the coal-rock particles in the area far from the drum initially increased and then decreased. The results are shown in Fig. 22. As a result, the maximum velocity and maximum displacement of the particles also initially increased and then decreased.

**Figure 23:**
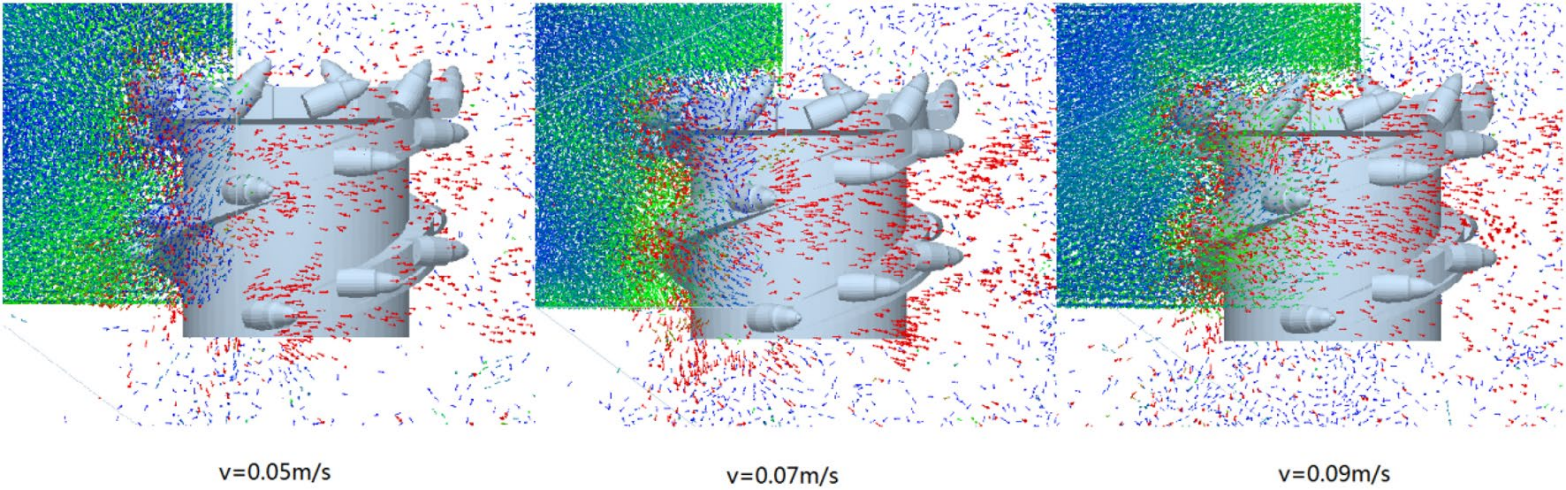
Velocity vector diagrams of the coal-rock particles under different traction speed conditions.

Similarly, the velocity vector diagram during the movement of the coal-rock particles was drawn. The velocity vector diagrams for the three working conditions and traction speeds of 0.05, 0.07, and 0.09 m/s were selected for analysis (Fig. 23).

As can be seen from Fig. 23, when the traction speed was 0.07 m/s, the number of coal particles that changed their initial velocity direction was the largest. By observing the coal-rock particles in the uncut coal wall, it was found that for an attraction speed of 0.07 m/s, a large proportion of the coal-rock particles in the coal wall to be cut exhibited movement trends, which was further conducive to the next coal falling process of the spiral drum. However, for traction speeds of 0.05 and 0.09 m/s, the movement trend of the uncut coal wall particles in the coal walls was not obvious.

The position coordinates of the coal-rock particles under different traction speeds were extracted (Table 6).

**Table 6:** Position coordinates of coal and rock particles under different traction speed conditions.

Working condition	Z1	X	Y	Z2	X	Y
C1	− 0.295845	0.5621477	0	− 0.357841	0	− 0.320243
C2	− 0.288636	0.5709411	0	− 0.371657	0	− 0.346511
C3	− 0.245156	0.5898954	0	− 0.293872	0	− 0.302312
C4	− 0.326225	0.6331205	0	− 0.364990	0	− 0.332215
C5	− 0.325414	0.6175422	0	− 0.360769	0	− 0.331459

By solving the parabolic equation of the coal-rock particle falling-coal trajectory using the parameters in Table 6, the following results were obtained:

24 $$\left\{ \begin{gathered} Z_{1} = - 2.3217x_{1}^{2} - 0.5933y_{1}^{2} + 0.4 \hfill \\ Z_{2} = - 2.1126x_{2}^{2} - 6.4268y_{2}^{2} + 0.4 \hfill \\ Z_{3} = - 1.8540x_{3}^{2} - 7.5922y_{3}^{2} + 0.4 \hfill \\ Z_{4} = - 1.8118x_{4}^{2} - 6.9313y_{4}^{2} + 0.4 \hfill \\ Z_{5} = - 1.7735x_{5}^{2} - 8.3264y_{5}^{2} + 0.4 \hfill \\ \end{gathered} \right.$$

**Figure 24:**
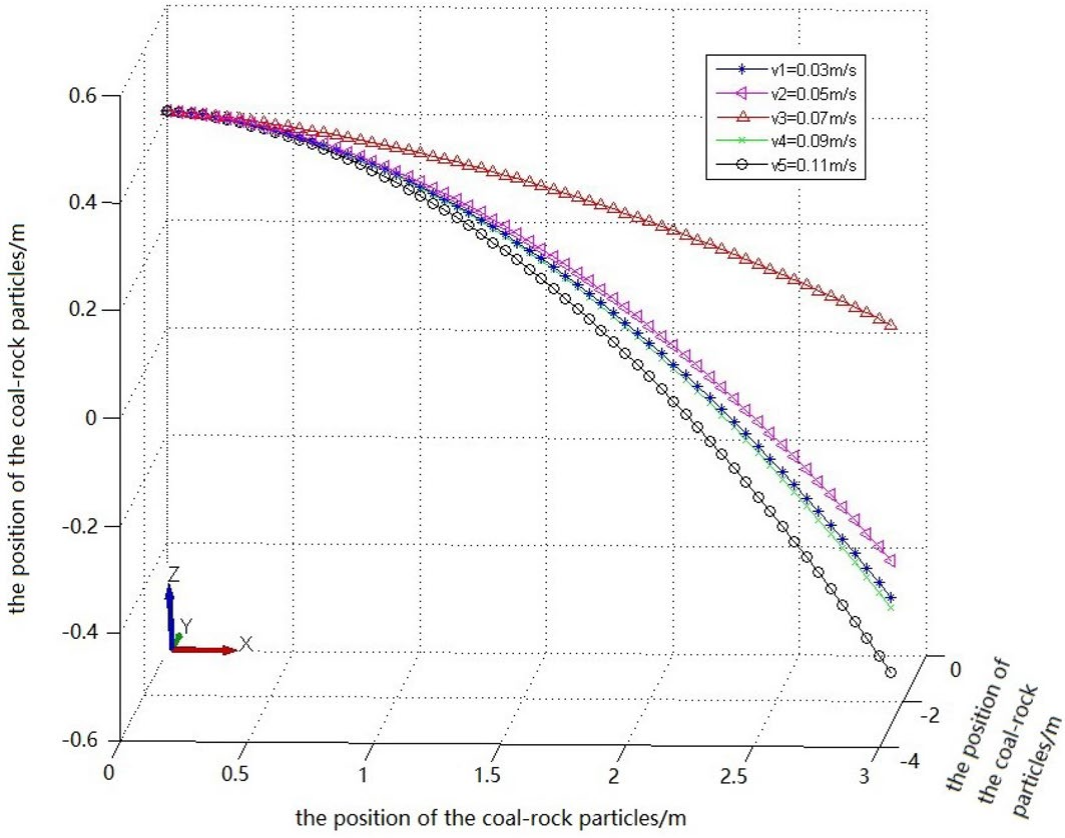
The trajectory of falling-coal in different traction speed.

The falling-coal trajectory curves under different traction speeds were drawn using MATLAB (Fig. 24).

It can be seen from the curve in Fig. 24 that as the traction speed increased, the opening of the parabolic curve of the falling-coal trajectory initially increased and then decreased. That is, with increasing traction speed, the distance that coal-rock particles were thrown initially increased and then decreased. This is consistent with the conclusion based on the velocity nephogram of the coal-rock particles, which verifies the correctness of the trajectories of the coal-rock particles with different traction speeds.

### Determination of cutting parameters under optimal coal falling trajectory conditions based on full factor experimental method

#### Setting of experimental scheme

Based on the full factor experimental method, working conditions A2, A3, A4, B2, B3, B4, C2, C3, and C4 were selected for full factor experiments. The cutting parameters under the optimal coal falling trajectory of coal and rock particles were determined through statistical analysis of the coal loading rate. The experimental scheme is presented in Table 7.

**Table 7:** Full factor experimental conditions.

Experimenttype	Experiment number	The cutting depth/*b*(mm)	Rotating speed/*n*(rad/s)	Traction speed/*v*(m/s)
A	1	480	8.5	0.05
	2	480	9.5	0.05
	3	480	10.5	0.05
B	4	480	8.5	0.07
	5	480	9.5	0.07
	6	480	10.5	0.07
C	7	480	8.5	0.09
	8	480	9.5	0.09
	9	480	10.5	0.09
D	10	530	8.5	0.05
	11	530	9.5	0.05
	12	530	10.5	0.05
E	13	530	8.5	0.07
	14	530	9.5	0.07
	15	530	10.5	0.07
F	16	530	8.5	0.05
	17	530	9.5	0.09
	18	530	10.5	0.09
G	19	580	8.5	0.05
	20	580	9.5	0.05
	21	580	10.5	0.05
H	22	580	8.5	0.07
	23	580	9.5	0.07
	24	580	10.5	0.07
I	25	580	8.5	0.09
	26	580	9.5	0.09
	27	580	10.5	0.09

#### Calculation process of coal loading rate statistics

**Figure 25:**
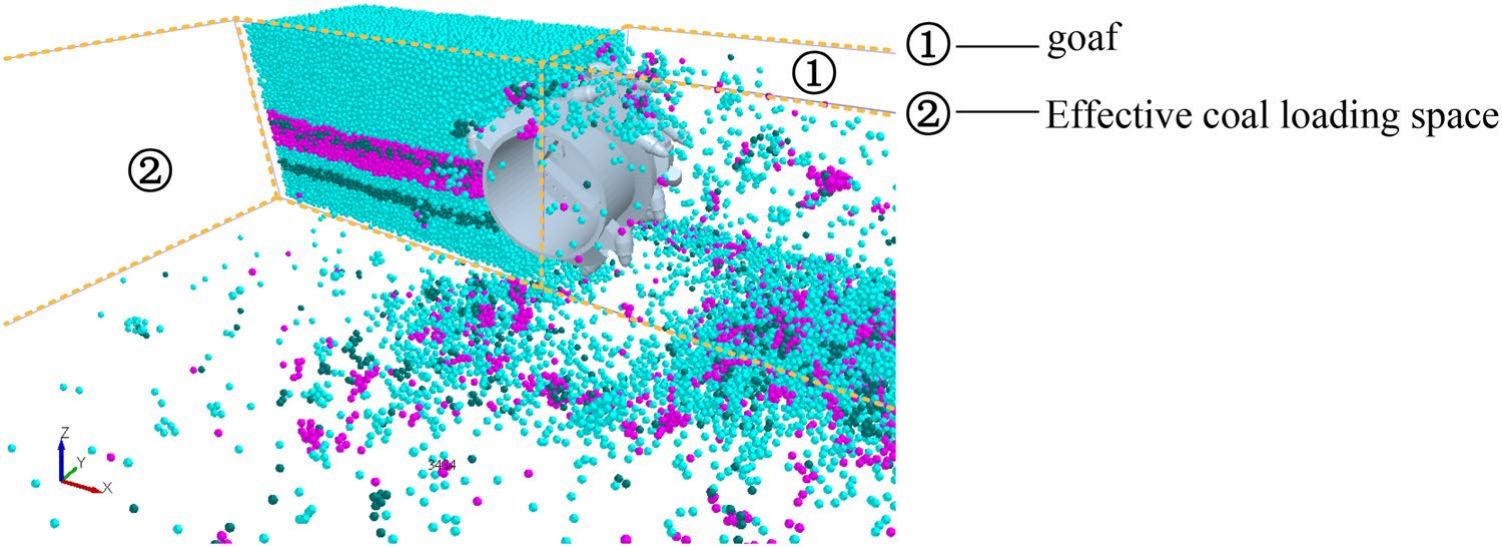
Effective coal loading area division.

The coal falling trajectory of the coal and rock particles directly affects the coal loading rate of the drum. When the coal and rock particles are concentrated in the effective coal loading area, the coal loading rate inevitably increases; otherwise, the coal loading rate decreases. The coal mining face was divided into goaf and effective coal loading areas through post-processing in EDEM (Fig. 25).

The difference in the densities of the rock particles and coal particles in the coal wall results in different qualities, and thus, the traditional quality statistics method of determining the coal loading rate is no longer accurate. In this study, the shapes of the rock particles and coal particles were the same. Therefore, calculating the coal loading rate by counting the number of particles in the goaf and the effective coal loading is more accurate. In the post-processing in EDEM, the corresponding statistical region is established. The number of particles in the goaf and the effective coal loading area for 27 groups of experiments were statistically analyzed. The results are presented in Table 8.

**Table 8:** Coal loading rate statistics.

Experiment type	working condition	Number of particles in goaf	Number of particles in the coal loading space	Coal loading rate/s(%)
A	1	2859	1860	39.42
	2	2910	2003	41.01
	3	2914	2115	42.06
B	4	4773	3154	39.79
	5	4613	3379	42.28
	6	4394	3448	43.97
C	7	6742	3940	36.88
	8	6314	4542	41.84
	9	6291	4650	42.50
D	10	3459	1759	33.71
	11	3434	1941	36.11
	12	3312	2110	38.92
E	13	5433	3039	35.87
	14	5455	3510	39.15
	15	5181	3434	39.86
F	16	7465	4087	35.38
	17	7233	4528	38.50
	18	7313	4678	39.01
G	19	3804	1858	32.82
	20	3776	1995	34.57
	21	3618	2229	38.12
H	22	5923	3067	34.12
	23	5810	3410	36.98
	24	6010	3770	38.55
I	25	8396	4285	33.79
	26	8455	3476	29.13
	27	8128	4851	37.38

#### Analysis of results

According to the data presented in Table 8, when the cutting parameters of the drum are a traction speed of 0.07 m/s, a rotation speed of 10.5 rad/s, and a cutting depth of 480 mm, the coal loading rate of the drum is the highest (0.4397). Based on this, it was concluded that the optimal coal falling trajectory can be expressed as follows:

25 $$Z = - 0.6222x^{2} - 3.1231y^{2} + 0.4$$

**Figure 26:**
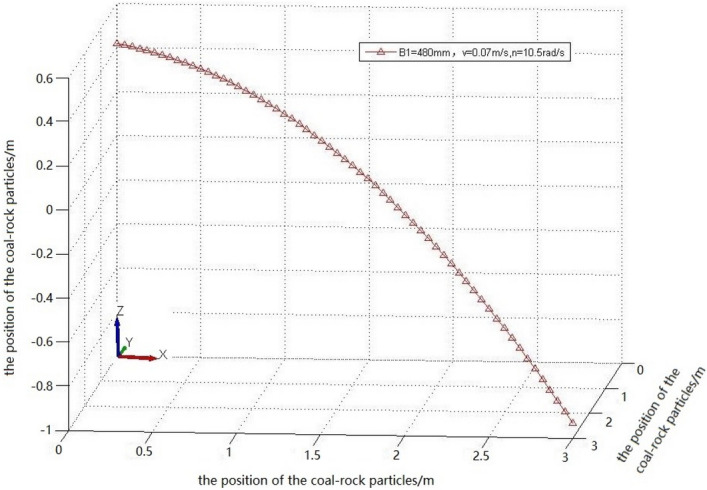
The optimal coal falling trajectory.

The optimal coal falling trajectory equation was fit, and the optimal coal falling trajectory is shown in Fig. 26.

## Analysis of strength and fatigue life of shearer spiral drum based on optimal cutting parameters

### Analysis of simulation results

**Figure 27:**
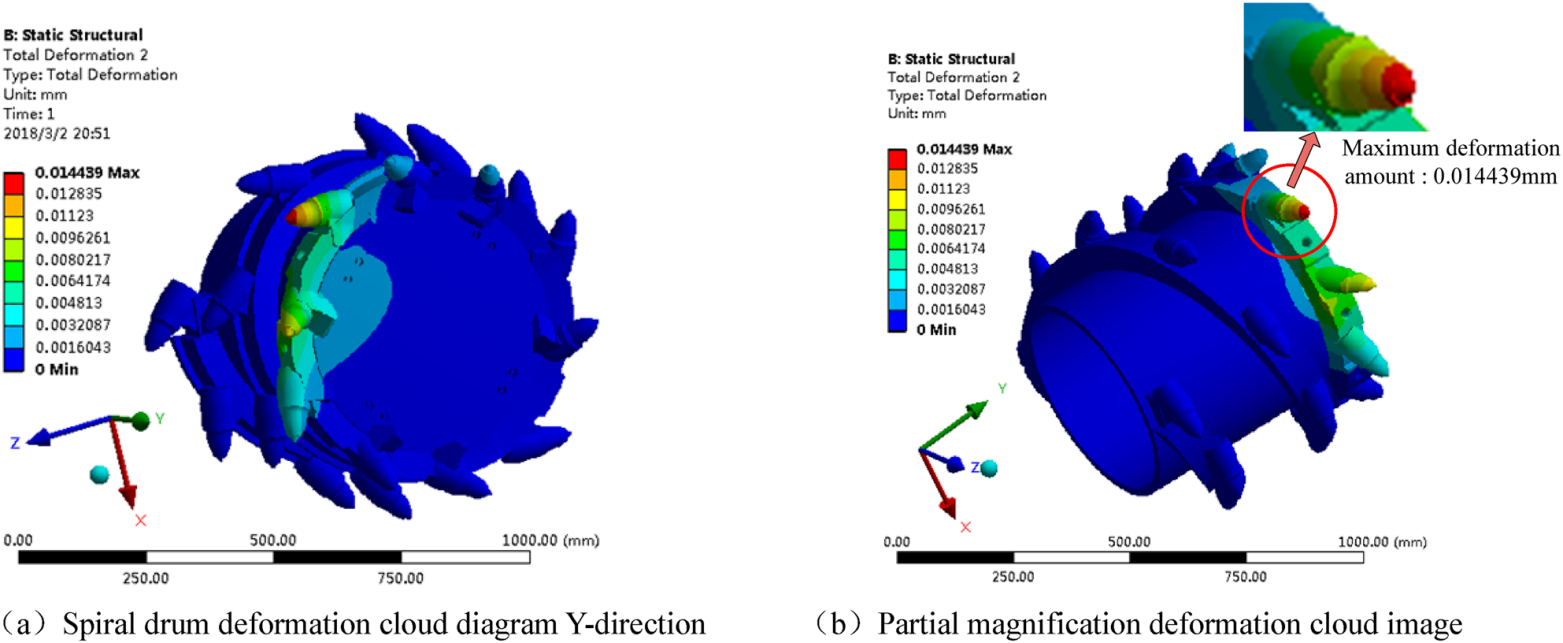
Spiral drum deformation cloud diagram.

Based on the EDEM-RecurDyn bidirectional coupling simulation results for the spiral drum and coal wall of the shearer under the optimal cutting parameters, the stress and deformation cloud map of the drum during the cutting of the coal wall with gangue were created via RecurDyn post-processing (Fig. 27).

As shown in Fig. 27a, based on the Y-direction in the cloud image, it can be seen that the left half of the end plate (which is participating in the cutting of the coal wall) has undergone local deformation, with a deformation amount of 0.004813 mm. As can be seen from Fig. 27b, the cutting pick on the end plate participating in the cutting of the coal wall has undergone deformation at this time. This is because the cutting pick on the end plate is located at the deepest point on the cutting coal wall, and its working environment is the most harsh, resulting in the greatest deformation. By observing the movement process of the drum in EDEM (Fig. 27), the working state of the cutting pick at the end plate is cutting hard gangue, so its deformation is the most obvious. The maximum deformation occurs at the tip of the tooth, with a deformation of 0.014439 mm.

**Figure 28:**
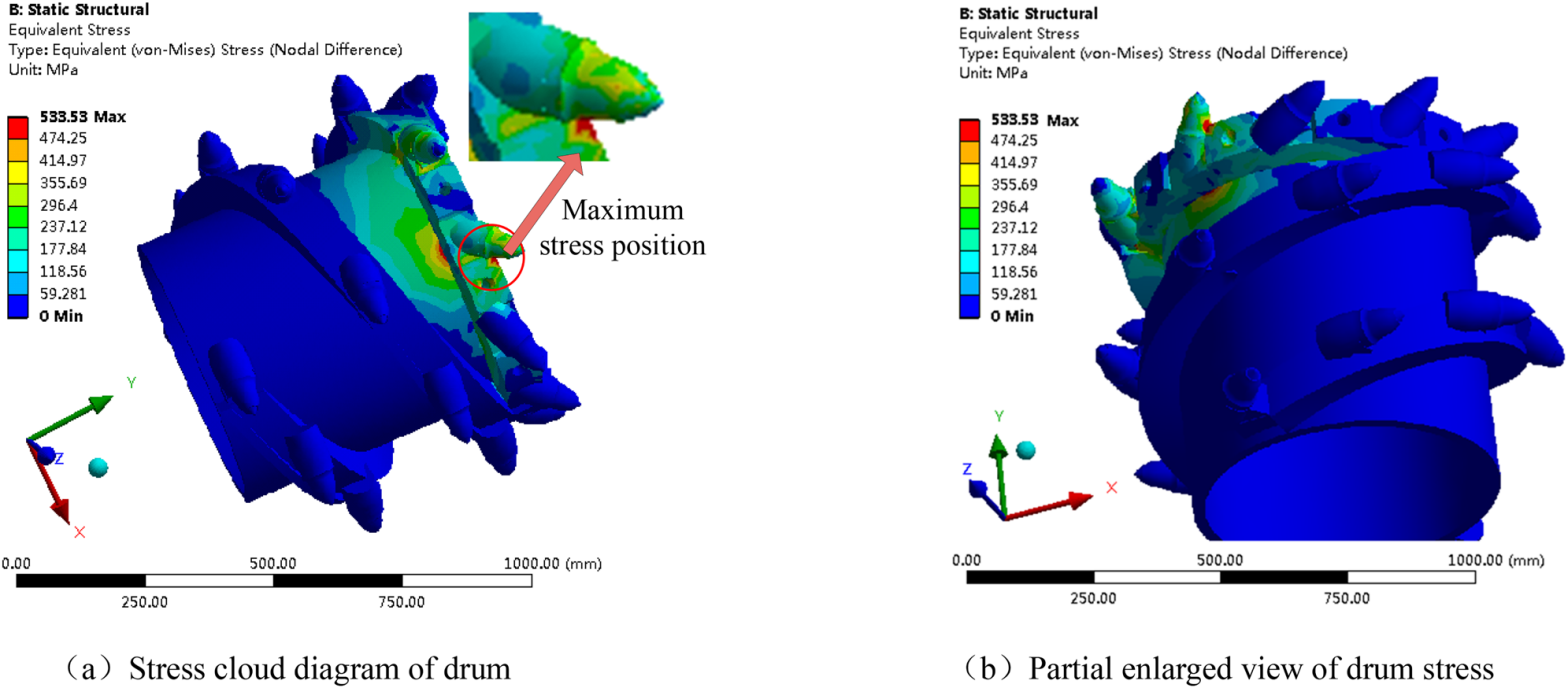
Stress cloud diagram of drum.

Based on the stress deformation analysis of the above-mentioned drum during the cutting of the coal wall, the load of the pick with the highest stress deformation was extracted, and its stress cloud diagram is shown in Fig. 28.

As can be seen from Fig. 28, during the cutting process, the part with the highest stress is at the root of the tooth seat, with a maximum stress of 533.53 MPa. As can be seen from the deformation cloud map of the spiral drum, its maximum deformation occurs on the cutting teeth that are cutting hard gangue, with a deformation of 0.014439 mm. However, compared with the overall structural size of the pick, this amount of deformation has approximately no impact on the drum. The stress of the working pick tooth seat is mainly concentrated at the root of the tooth seat, which is determined by the structure of the tooth seat of the pick. The material yield limit of the tooth seat is 1080 MPa, which is greater than the maximum equivalent stress value of 533.53 MPa generated by the tooth seat under a static force. Therefore, the lowest factor of safety of the drum is calculated to be 2.024. Even if the yield limit at the weld seam is (0.8–0.9) × 1080 MPa, the minimum factor of safety of the drum is 1.62, which also meets the safety requirements.

**Figure 29:**
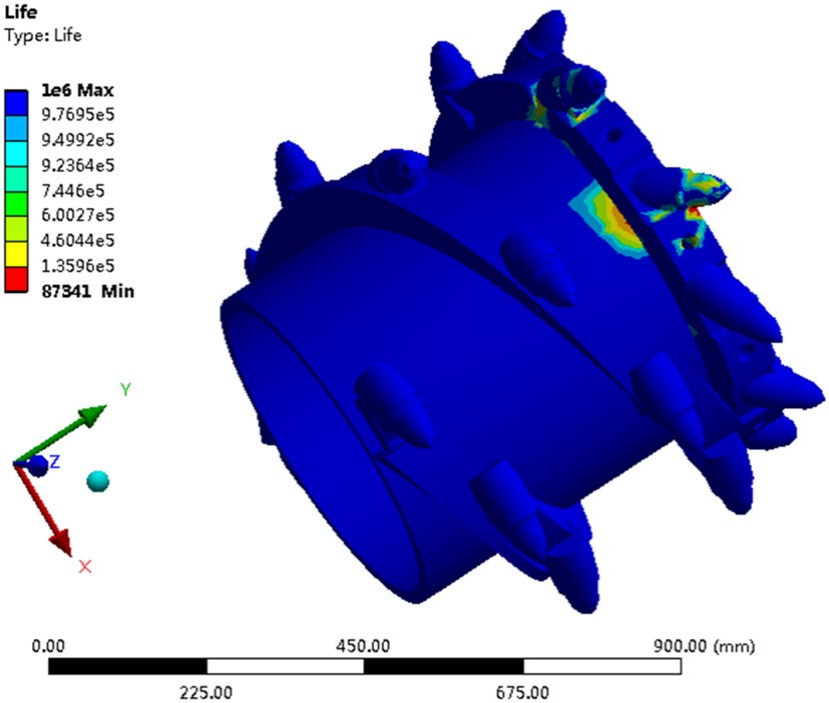
Cloud diagram of fatigue life of the highest loaded pick.

Considering the safety of the drum during operation, we predicted its fatigue life. The stress fatigue life of the drum was calculated through RecurDyn post-processing. The results are shown in Fig. 29.

As can be seen from Fig. 29, for the entire drum, the root of the tooth seat of the pick on the end plate that is participating in cutting the gangue has the lowest fatigue life, with a value of 87,341 cycles, which meets the usage requirements. The analysis results of the comprehensive equivalent stress, deformation, and fatigue life reveal that the material selection and structural design of the drum are safe and reliable during the working process for the cutting parameters selected under the condition of the maximum coal loading rate^40^.

### Validation experiment

#### Experimental platform renovation

To verify the safety and reliability of the designed spiral drum structure under the optimal cutting parameters, experimental testing was conducted on the cutting process of the spiral drum of the coal mining machine in a large industrial and mining equipment key laboratory. Due to the limitations of the laboratory space, based on the existing mining equipment in the laboratory and the similarity theory, the existing coal mining machine cutting the coal and rock experimental platform was renovated.

The drum diameter, drum rotation speed, traction speed, force, torque, cutting power, vibration acceleration, density, and strength were selected as similar parameters. During the construction process of the experimental platform, the MLT (quality system) dimensional analysis method was used to establish a dimensional table for the various parameters (Table 9).

**Table 9:** Parameter dimension table.

Parameter	*M*	*L*	*T*
Drum diameter/*D*(mm)	0	1	0
Drum rotating speed/*n* (r min ^1^)	0	0	− 1
Traction speed/*v*_*q*_(m·min-1)	0	1	− 1
Density/*ρ*(kg m^−3^)	1	− 3	0
Strength/*σ*(MPa)	1	− 1	− 2
Force/*F*(N)	1	1	− 2
Moment/*T*(N·m)	1	2	− 2
Cutting power/*P* (kw)	1	2	− 3
Vibration acceleration/*a*(mm s^−32^)	0	1	−2

**Figure 30:**
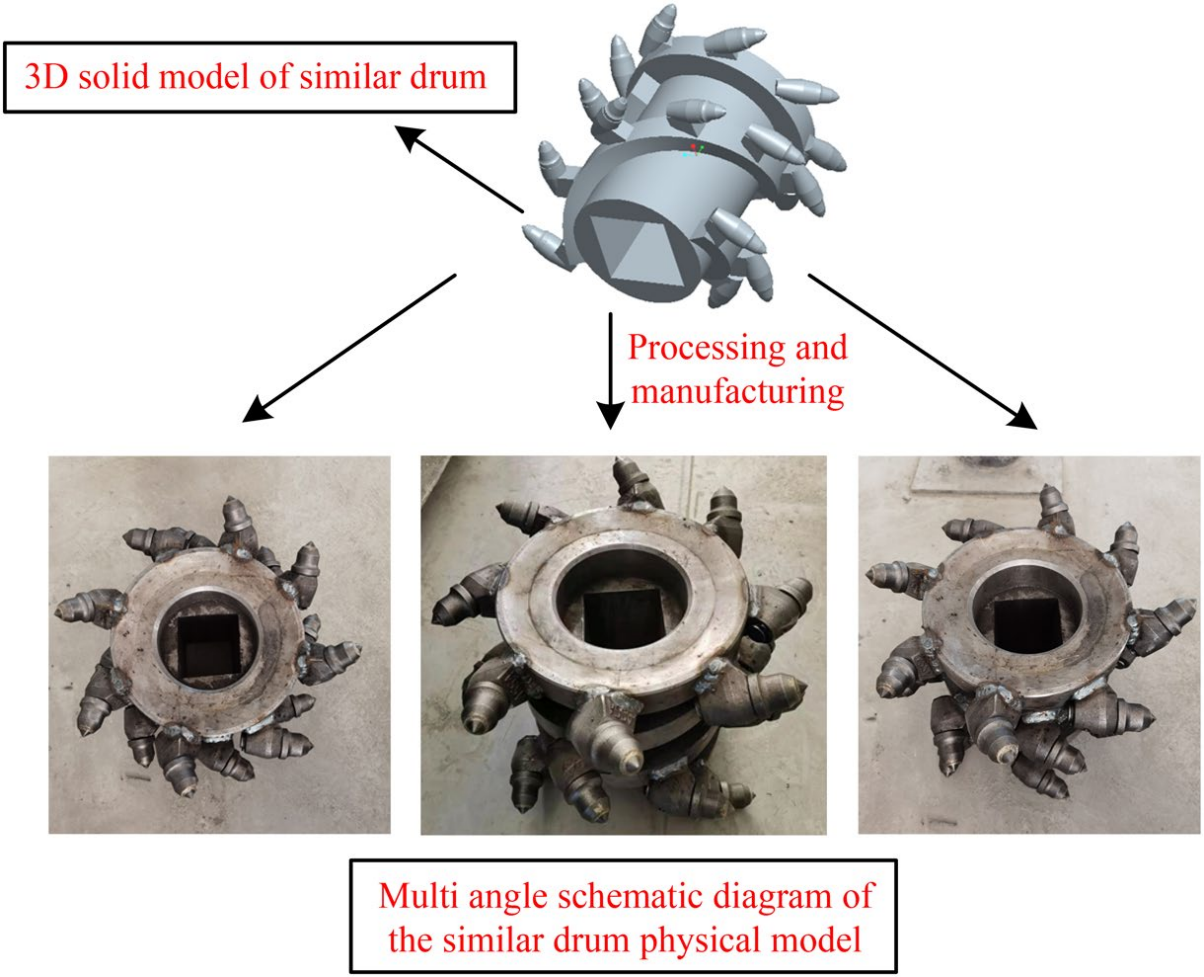
Drum simulation and solid model.

For the design of the similarity models, the determination of similarity coefficients is shown in Table 10. The 3-D solid model of the drum designed based on the principle of similarity and the actual model processed are shown in Fig. 30.

**Table 10:** Similarity coefficient. Significant values are in italics.

Similarity coefficient	Numerical value
*C*_*L*_	*K*_*1*_ = *0.5*
*C*_*n*_	*K*_2_ = 1.2
*C*_*ρ*_	*K*_3_ = 1
*C*_*D*_	*K*_1_
*C*_*v*_	*K*_1_*K*_2_
*C*_*σ*_	*K*_1_^2^*K*_2_^2^*K*_3_
*C*_*F*_	*K*_1_^4^*K*_2_^2^*K*_3_
*C*_*T*_	*K*_1_^5^*K*_2_^2^*K*_3_
*C*_*P*_	*K*_1_^5^*K*_2_^3^*K*_3_
*C*_*a*_	*K*_1_*K*_2_^2^
*C*_*f*_	*K*_1_^2^*K*_2_^2^*K*_3_

#### Construction of a comprehensive experimental system and verification of spiral drum strength experiment

**Figure 31:**
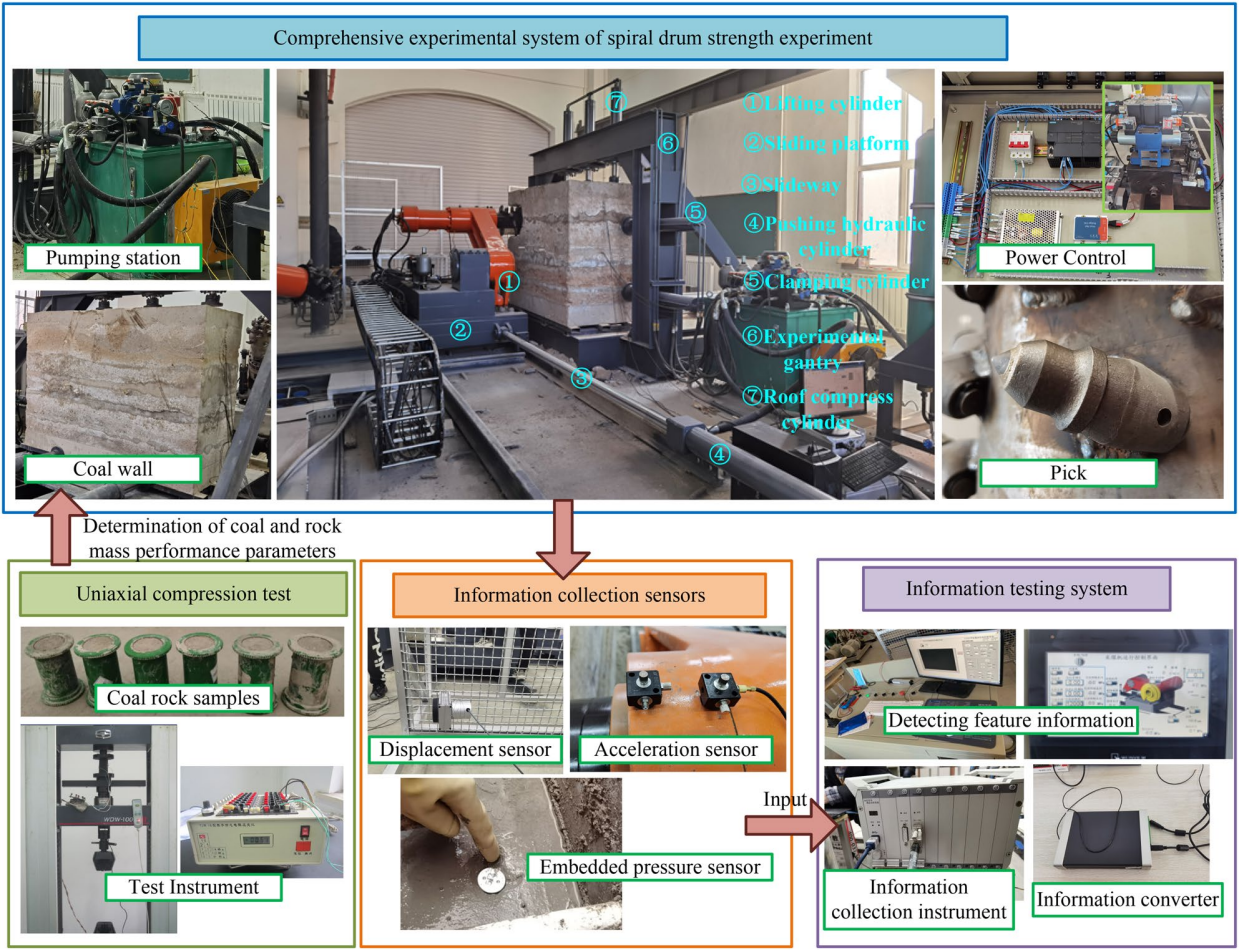
Comprehensive experimental system of spiral drum strength experiment.

**Figure 32:**
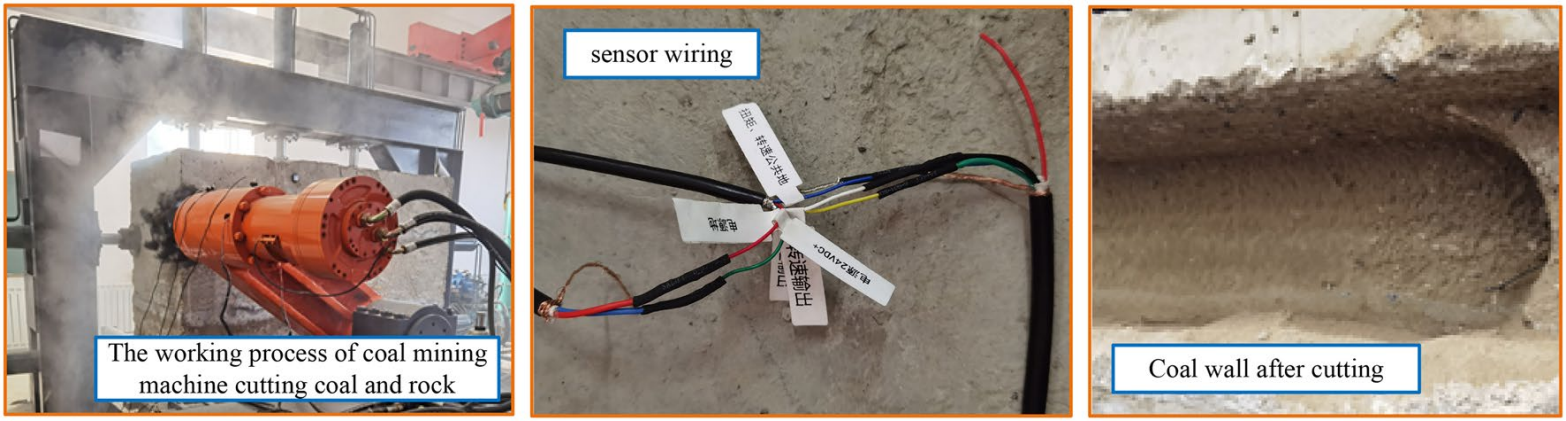
The process of the experiment.

The composition of the comprehensive experimental system for coal rock cutting is shown in Fig. 31. The implementation process of its validation experiment is shown in Fig. 32.

**Figure 33:**
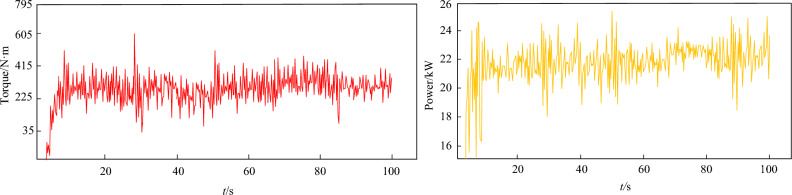
Test data.

The test data for the torque sensor after cutting under the optimal cutting parameters are shown in Fig. 33. As shown in Fig. 33, the average values of the torque and power of the spiral drum after smooth rotation are about 288 N·m and 22 kW, respectively. Based on the radius of the cutting drum, a similar reverse inverse calculation was performed on the torque of the drum obtained from the experiment and the average cutting resistance received. The error between the reverse inference results and the simulation results is 4.4, demonstrating that the cutting test system established using the principle of similarity has a high accuracy and can meet the experimental requirements.

**Figure 34:**
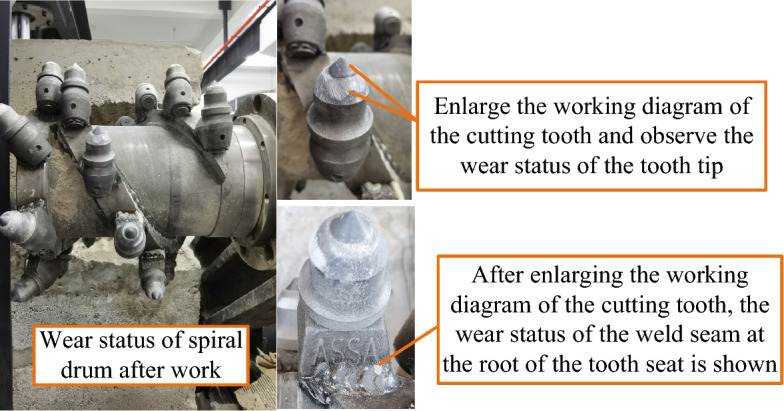
The drum wear status of the shearer.

After working for one cycle under the optimal cutting parameters, the drum wear status of the shearer is shown in Fig. 34. As can be seen from Fig. 34, after one cycle of operation, the surface of the shearer exhibited slight wear. This type of wear was mainly concentrated at the tip of the cutting tooth and the welding seam of the root of the tooth seat. However, this wear did not cause significant deformation of the drum, and the structure of the drum was safe and reliable.

### Industrial experimental verification

**Figure 35:**
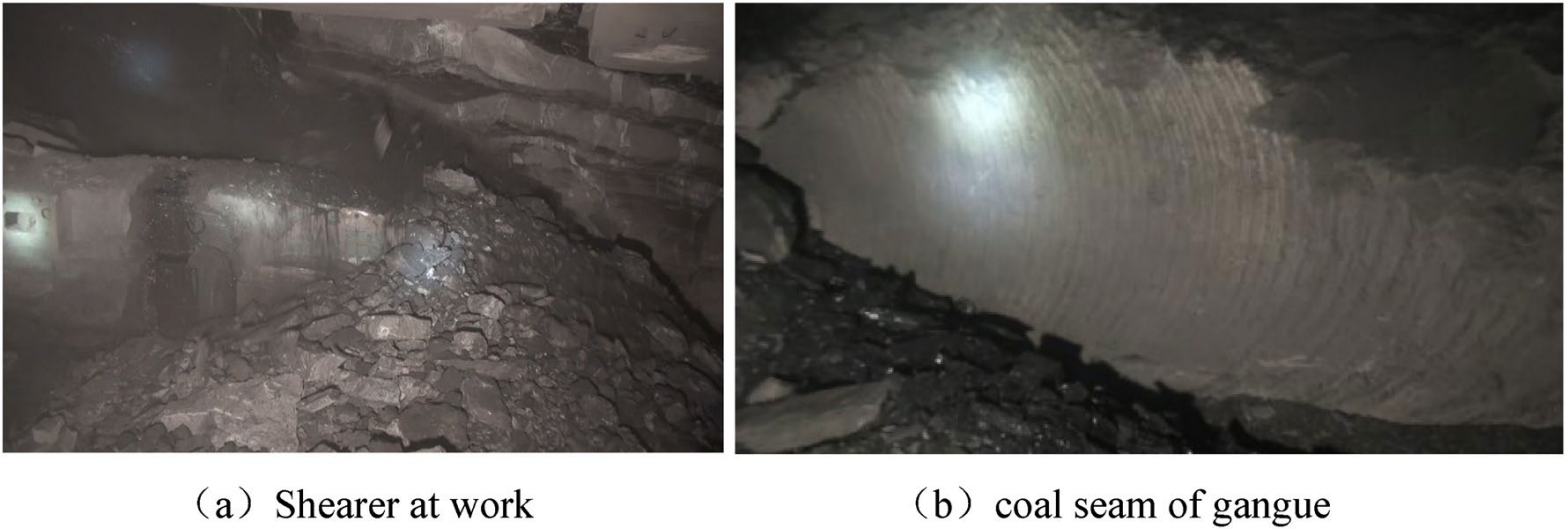
Underground experiment.

The MG2 × 55/250-BWD thin seam shearer spiral drum based on the optimization results was jointly designed by the research group, Datong Phillips Mining Machinery Manufacturing Co., Ltd., Yankuang Group, and other units. The spiral drum has been industrially tested in the 17th coal seam of the Yangcun Mine of the Yankuang Group and has been officially put into production (Fig. 35). Figure 35a shows the state of the coal mining machine during operation. Figure 35b shows the exposed gangue in the coal seam after being cut by the shearer. The actual measured average coal loading rate of the front drum of the MG2 × 55/250-BWD shearer was 46.31%. The shearer is currently being used in the Yanzhou Coal Industry Headquarters coal mine and it has achieved stable operation and has a safe and reliable structure.

## Conclusions

Through experimental verification, discrete element model of high-precision coal wall with complex gangues established in this article is closer to the physical properties of actual underground coal and rock than the traditional discrete element model.

Experimental results have shown that the virtual prototype model of the shearer cutting coal wall constructed based on bidirectional coupling method has a smaller error between the experimental results and the actual working state compared to the model constructed using unidirectional coupling method.

Based on the bidirectional coupling method, the coal falling trajectory of the spiral drum of the shearer were studied, and the results showed that: As the cutting depth and traction speed of the drum increase, the initial velocity of coal and rock particles shows a trend of first increasing and then decreasing, resulting in an increase and then decrease in the maximum velocity and maximum displacement during particle movement. As the drum rotating speed increases, the initial velocity of coal and rock particles shows an increasing trend, leading to a gradual increase in the maximum velocity and maximum displacement during particle movement.

Obtain the optimal coal falling trajectory through full factor experiments. Perform strength analysis on the drum based on the parameters of the drum under the optimal coal falling trajectory working condition. The maximum deformation of the drum occurs at the cutting tooth position where the hard gangues is being cut. The stress of the tooth seat is mainly concentrated at the root of the tooth seat, with a maximum equivalent stress value of 533.53 MPa, which is less than the yield limit. Therefore, the material selection and structural design of the drum are safe and reliable. For the entire drum, the root life of the tooth seat of the cutting teeth on the end plate that is participating in the cutting of gangue is the lowest, with a value of 87,341 cycles, which meets the usage requirements.

Based on the similarity theory, an experimental platform was constructed to verify the drum structure designed and analyzed, and the results showed that the drum structure was safe and reliable. After being put into production, it runs stably with an average coal loading rate of 46.31%. This provides new theories and methods for designing spiral drums for structural evolution.

## Data Availability

All data generated or analysed during this study are included in this published article.
